# Supplementary material for: Thor: a platform for cell-level investigation of spatial transcriptomics and histology
Source: Nat Commun. 2025 Aug 5;16:7178. doi: 10.1038/s41467-025-62593-1 (PMC12325965; doi:10.1038/s41467-025-62593-1)
Supplement: Supplementary file 1 — Supplementary Information [file 41467_2025_62593_MOESM1_ESM.pdf]

Supplementary information

Thor: a platform for cell-level investigation of spatial transcriptomics and histology

Pengzhi Zhang[1,2,3,4,#], Weiqing Chen[5,#], Tu N Tran[1,2,3,4], Minghao Zhou[6], Kaylee N. Carter[4], Ibrahem Kandel[1,2,3,4], Shengyu Li[1,2,3,4], Xen Ping Hoi[7,8,9], Yuxing Sun[10], Li Lai[4], Keith Youker[4], Qianqian Song[6], Yu Yang[11], Fotis Nikolos[7,8], Zejuan Li[12,13], Keith Syson Chan[7,8], John P. Cooke[2,3,4], Guangyu Wang[1,2,3,4,5*]

1. Center for Bioinformatics and Computational Biology, Houston Methodist Research Institute, Houston, TX, 77030, USA
2. Center for RNA Therapeutics, Houston Methodist Research Institute, Houston, TX, 77030, USA
3. Department of Cardiothoracic Surgery, Weill Cornell Medicine, Cornell University, New York, NY, 10065, USA
4. Department of Cardiovascular Sciences, Houston Methodist Research Institute, Houston, TX, 77030, USA
5. Department of Physiology, Biophysics & Systems Biology, Weill Cornell Graduate School of Medical Science, Weill Cornell Medicine, Cornell University, New York, NY, 10065, USA
6. Department of Health Outcomes and Biomedical Informatics, University of Florida, Gainesville, FL, 32610, USA
7. Department of Urology, Houston Methodist Research Institute, Houston, TX, 77030, USA
8. Spatial Omics Core, Neal Cancer Center, Houston Methodist Research Institute, Houston, TX, 77030, USA
9. Graduate Program in Biomedical Sciences, Cedars-Sinai Medical Center, Los Angeles, CA, 90069, USA
10. School of Computer Science, Georgia Institute of Technology, Atlanta, GA, 30308, USA
11. Department of Pathology, Immunology and Laboratory Medicine, College of Medicine, University of Florida, Gainesville, FL, 32608, USA
12. Department of Pathology and Genomic Medicine, Houston Methodist Hospital, Houston Methodist Research Institute, Houston, TX 77030, USA
13. Weill Cornell Medical College, New York, NY 10065, USA

# Those authors contributed equally to the work.
* Corresponding author: gwang2@houstonmethodist.org

Table of Contents

Supplementary Figures 2

Supplementary Note 1 35

Supplementary Figures

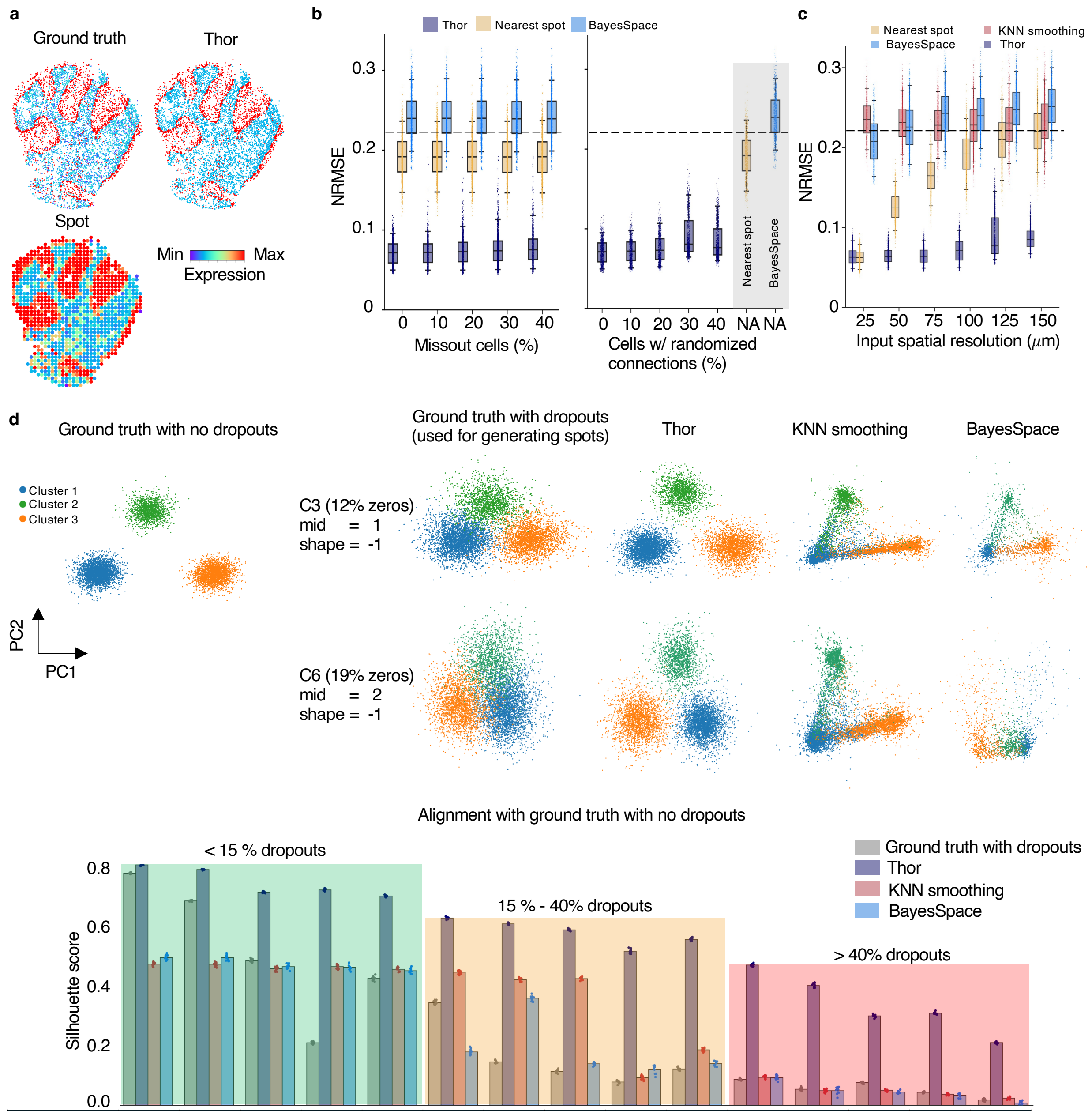

Condition	C1	C2	C3	C4	C5	C6	C7	C8	C9	C10	C11	C12	C13	C14	C15
**mid**	1	1	1	2	2	2	3	3	3	4	4	4	5	5	5
**shape**	-5	-2	-1	-5	-2	-1	-5	-2	-1	-5	-2	-1	-5	-2	-1
**drop %**	6.6	8.1	12	9.2	12.5	18.9	17.2	22.8	29.7	35.3	41.4	44.2	59.5	62.9	61.3

Supplementary Figure 1: Thor performance on simulation data. (a) Representative expression profiles of simulated spot-resolution, the ground truth, and Thor-predicted single-cell data. (b) Thor performance on data with cell missouts or perturbations in cell-cell network. The nearest spot method maps the expression of the closest spot to the cell; the subspot-level gene expression from BayesSpace is mapped to the identified cells using nearest cell neighbors. (c) Thor's performance on data with different spot sizes. (d) Thor imputes gene expression with technical dropouts. The error bars for the mean Silhouette coefficients are omitted as they are too small to visualize. n = 10 resamples in each condition. The colors in the PCA plots represent the ground truth cell type information. "Drop %" in the table is calculated as the ratio of zeros in the count matrix of the simulated scRNA-seq data. For the box plots, the middle line in the box plot, median; box boundary, interquartile range; whiskers, 5–95 percentiles; minimum and maximum, not indicated in the boxplot; gray dots, individual data points. Source data are provided in a Source Data file.

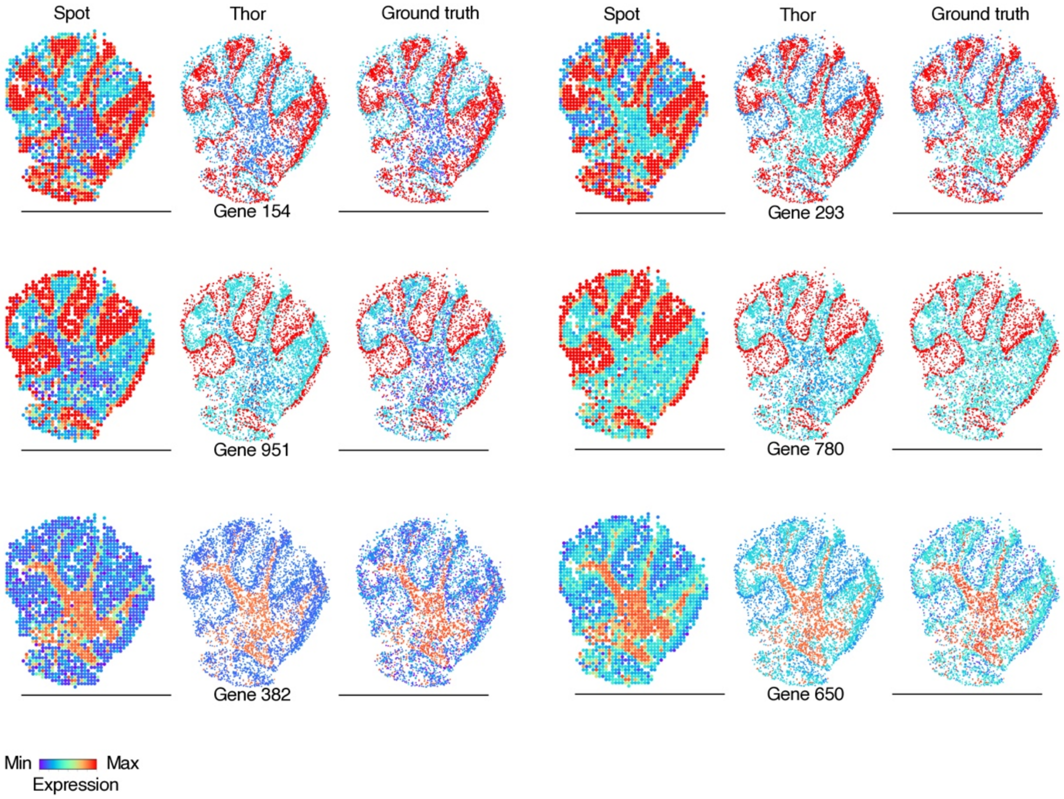

Supplementary Figure 2: Gene expression profiles in the simulated dataset. Spot-resolution data (spot separation: 100 μm), cell-resolution, and Thor-predicted single-cell data are visualized. Each row shows two marker genes for the same cell type.

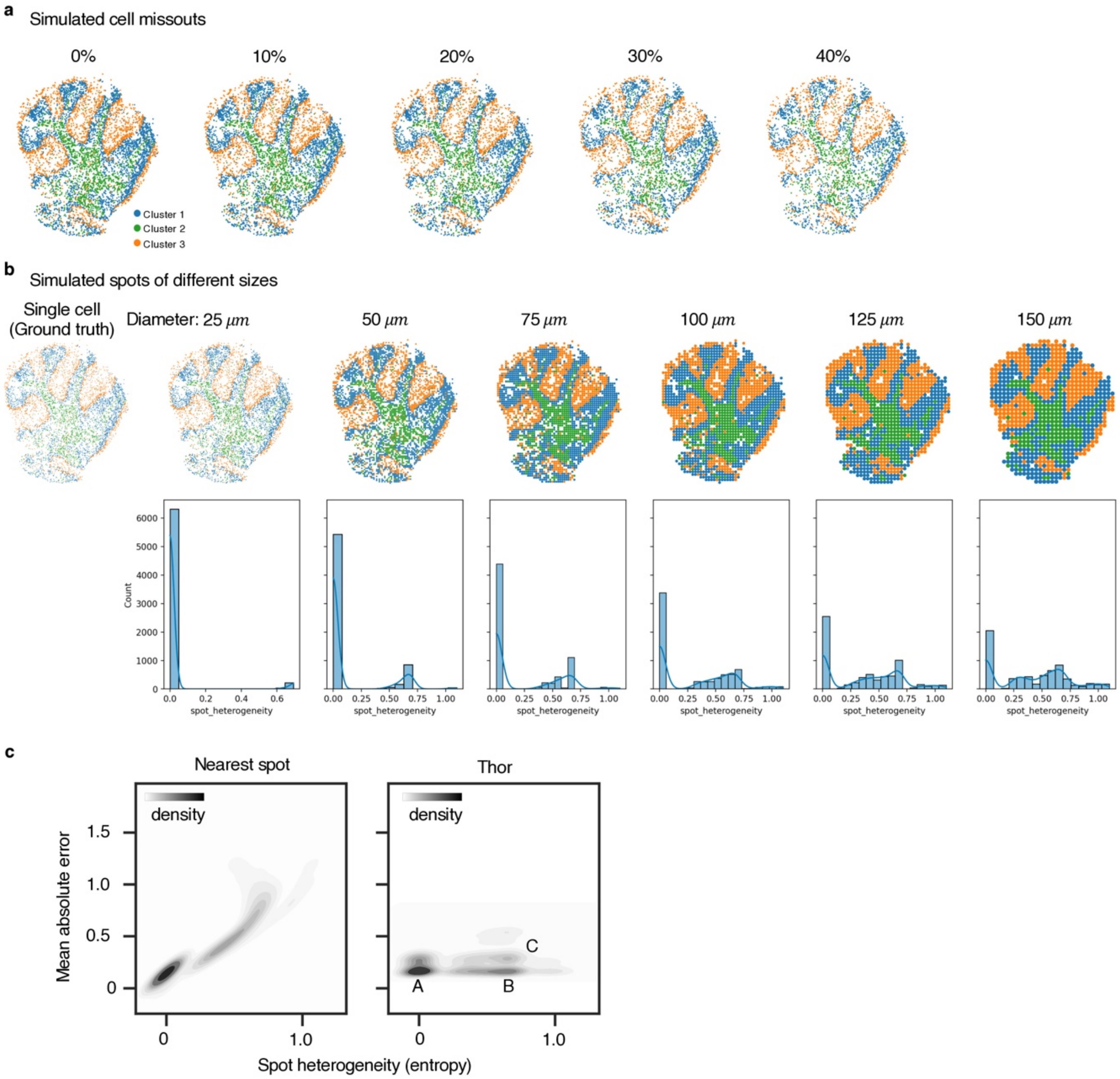

Supplementary Figure 3: Visualization of cell missouts and different spot sizes. (a) In the single-cell spatial transcriptome data, the miss-detection of cells from 10% to 40% randomly leads to sparser cell distribution. (b) The proportion of homogeneous spots (characterized by low spot heterogeneity scores) decreases as spot size increases. Spot heterogeneity is quantified using the Shannon entropy of cell type proportions within a spot. (c) Thor demonstrates robust performance across varying levels of spot heterogeneity. Mean absolute error (MAE) is calculated between Thor-predicted and ground-truth gene expression levels. 'A', 'B', 'C' mark one low-heterogeneity, and two high-heterogeneity clusters of the Thor data. Source data are provided in a Source Data file.

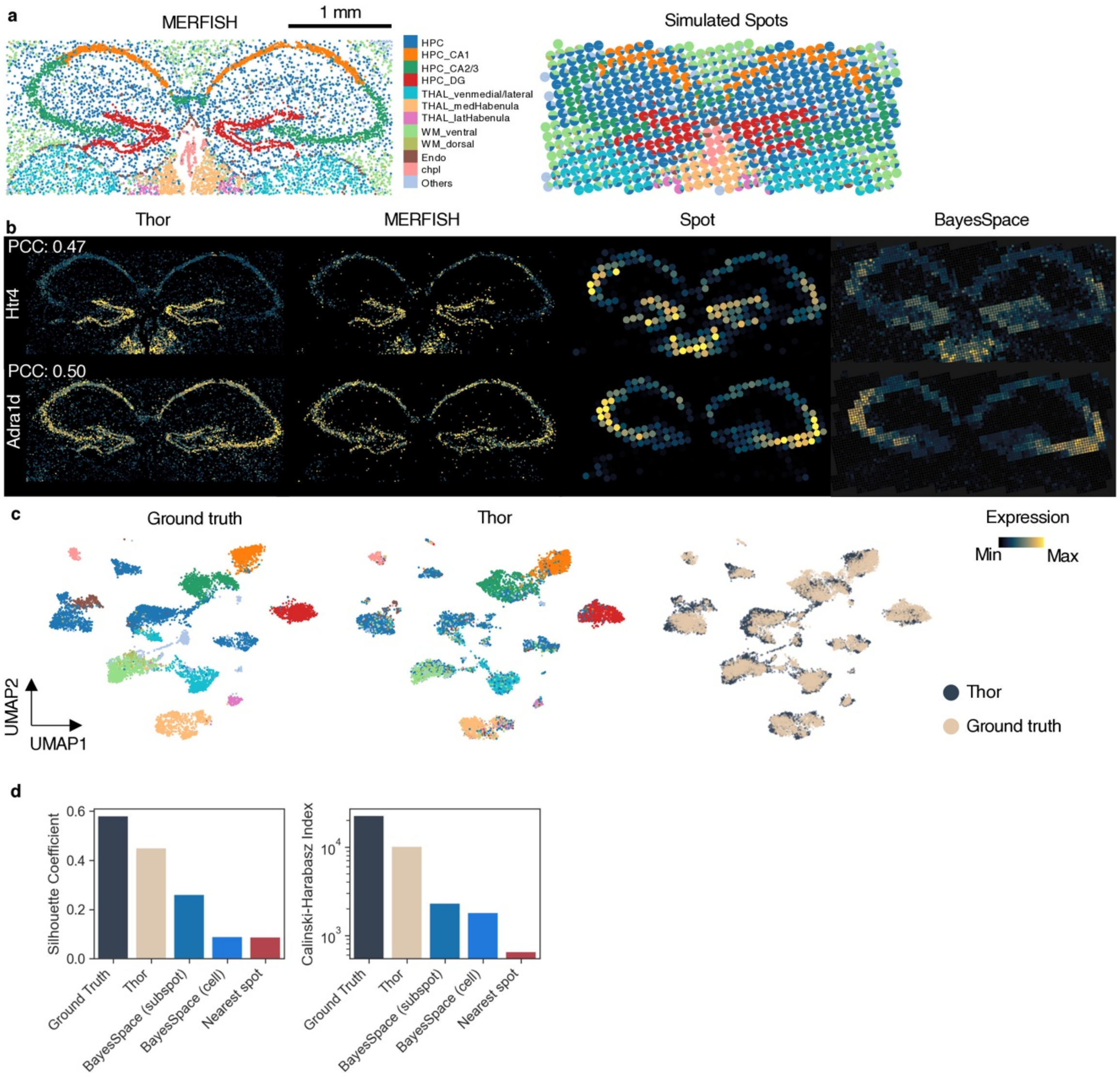

Supplementary Figure 4: Thor accurately predicts single-cell spatial gene expression from simulated spot-resolution gene expression in mouse hippocampus. (a) Cell-type distribution in the mouse hippocampus region from the MERFISH data (ground truth; left panel) and cell-type population in simulated spots (right panel). Spots in regions including the CA and DG, are composed of cells of diverse cell types. (b) Expression patterns of representative genes inferred by Thor (left panel), alongside the ground-truth MERFISH data (second panel), spot data (third panel), and subspot data (right panel). Pearson correlation coefficients are provided. (c) Clusters of the Thor-inferred *in silico* cells overlap well with the ground-truth cell clusters. (d) Quantitative evaluation of cell clustering. The Silhouette coefficient and Calinski-Harabasz index are calculated based on the embeddings and the ground truth cell annotations. For BayesSpace, as the native output is sub-spot level gene expression, both the sub-spot level and cell-level metrics are considered (mapping the closest sub-spots to the cells). n = 1 sample. Source data are provided in a Source Data file.

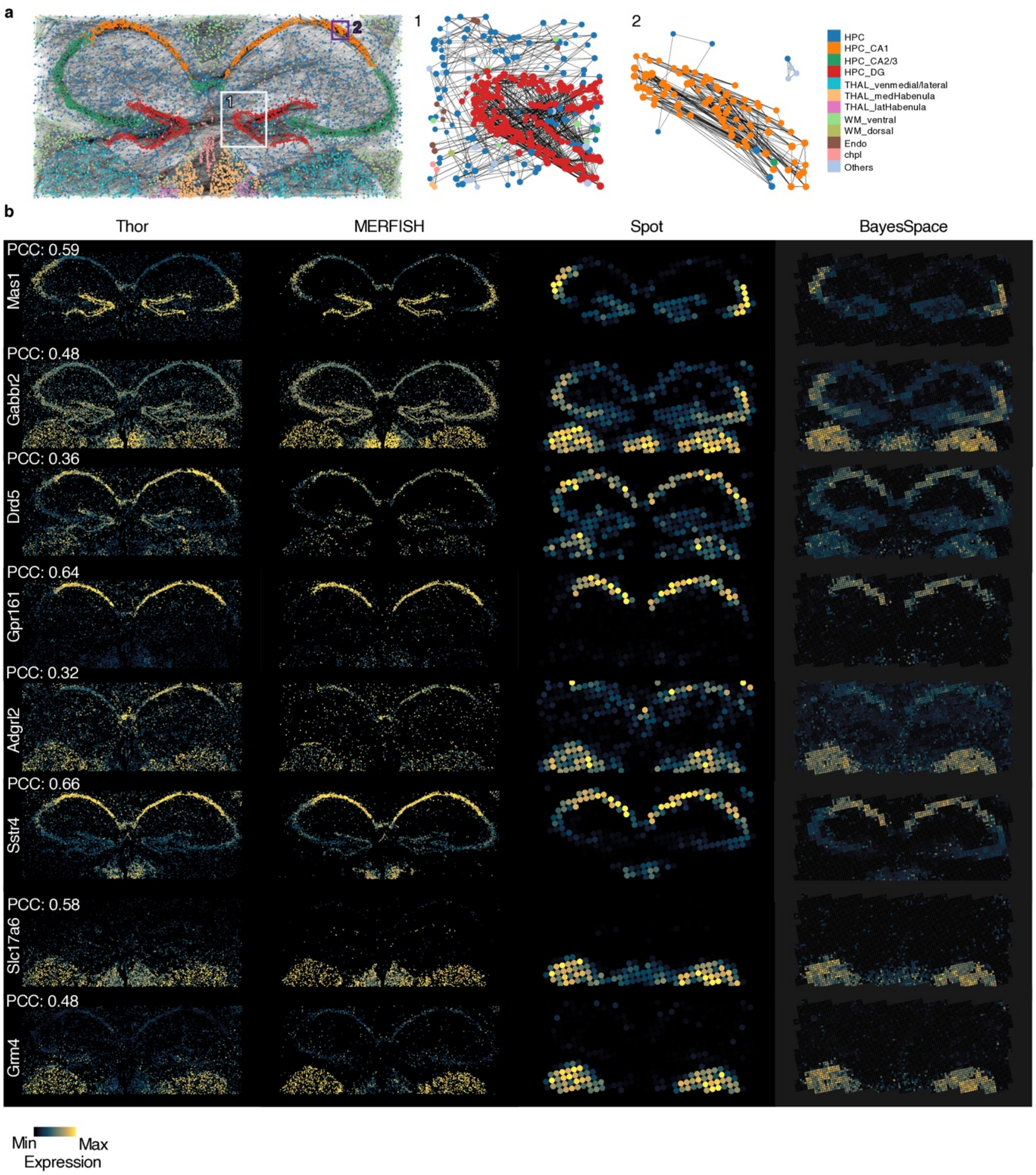

Supplementary Figure 5: Visualization of cell-cell network and predicted gene expression in the simulated mouse cerebellum data. (a) In the analysis of this simulated dataset, Thor connects similar cells based on location and image features. Cells of similar types are interconnected (note: cell type information is not used in constructing the cell-cell network). (b) Thor refines gene expression in various regions of the mouse hippocampus, demonstrated by a few selected genes inferred by Thor (left panel), alongside the ground-truth MERFISH data (second panel), spot data (third panel), and subspot data (right panel). Pearson correlation coefficients are provided.

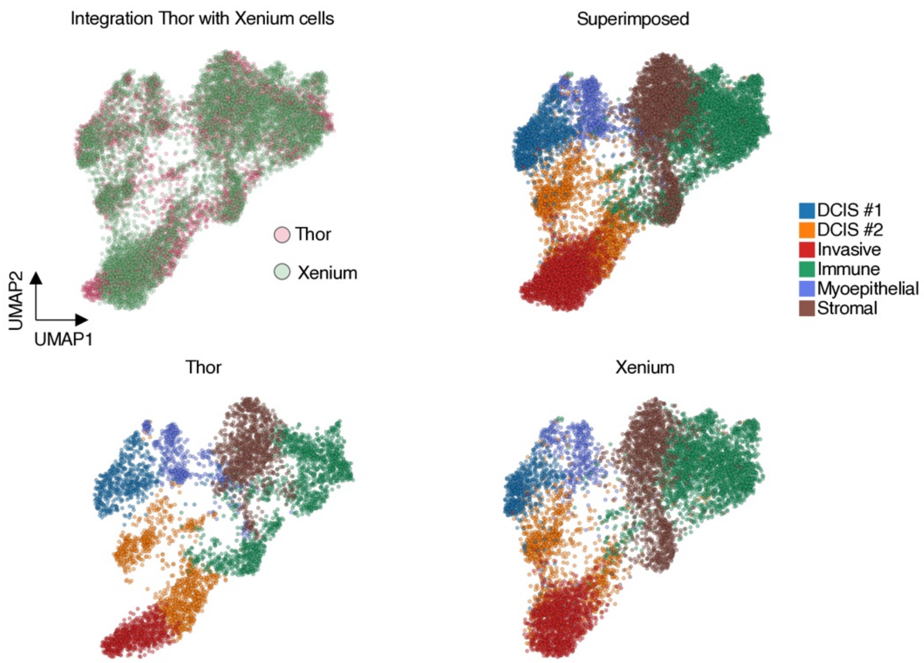

Supplementary Figure 6: UMAP embeddings of the Thor and Xenium integrated cells colored by modalities and by cell types. The embeddings were obtained from the PCA space by integration with harmonypy.

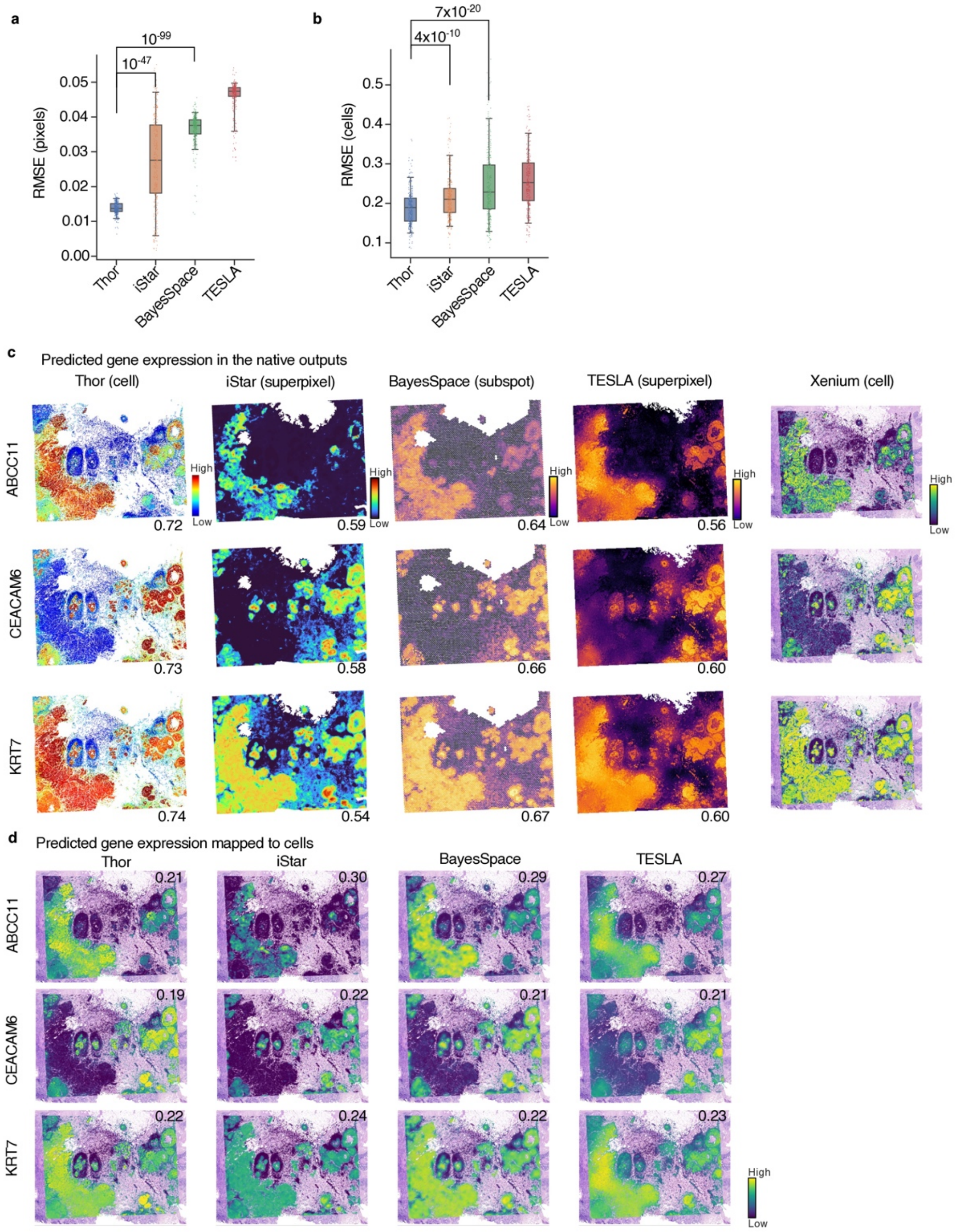

Supplementary Figure 7: Quantitative comparison between Thor and ST spatial resolution-enhancement tools. (a) Using image-level metrics, and (b) Using cell-level metrics. The box plots summarize the similarity across 306 genes included in the Xenium panel. The middle line in the box plot, median; box boundary, interquartile range; whiskers, 5–95 percentiles; minimum and maximum, not indicated in the boxplot. One-sided Mann-Whitney U test was performed to compare Thor with the two next-best-performing tools; corresponding p-values are shown in the plot. (c) Spatial profiles of representative genes inferred by Thor and other tools. Xenium gene expression profiles are provided for reference. The CW-SSIM scores are included. (d) Spatial profiles of representative genes inferred by Thor and other tools. The RMSE of Min-Max normalized cell level expressions are provided. Nearest cell/superpixel/subspot expression levels are mapped to the Xenium cell positions. Source data are provided in a Source Data file.

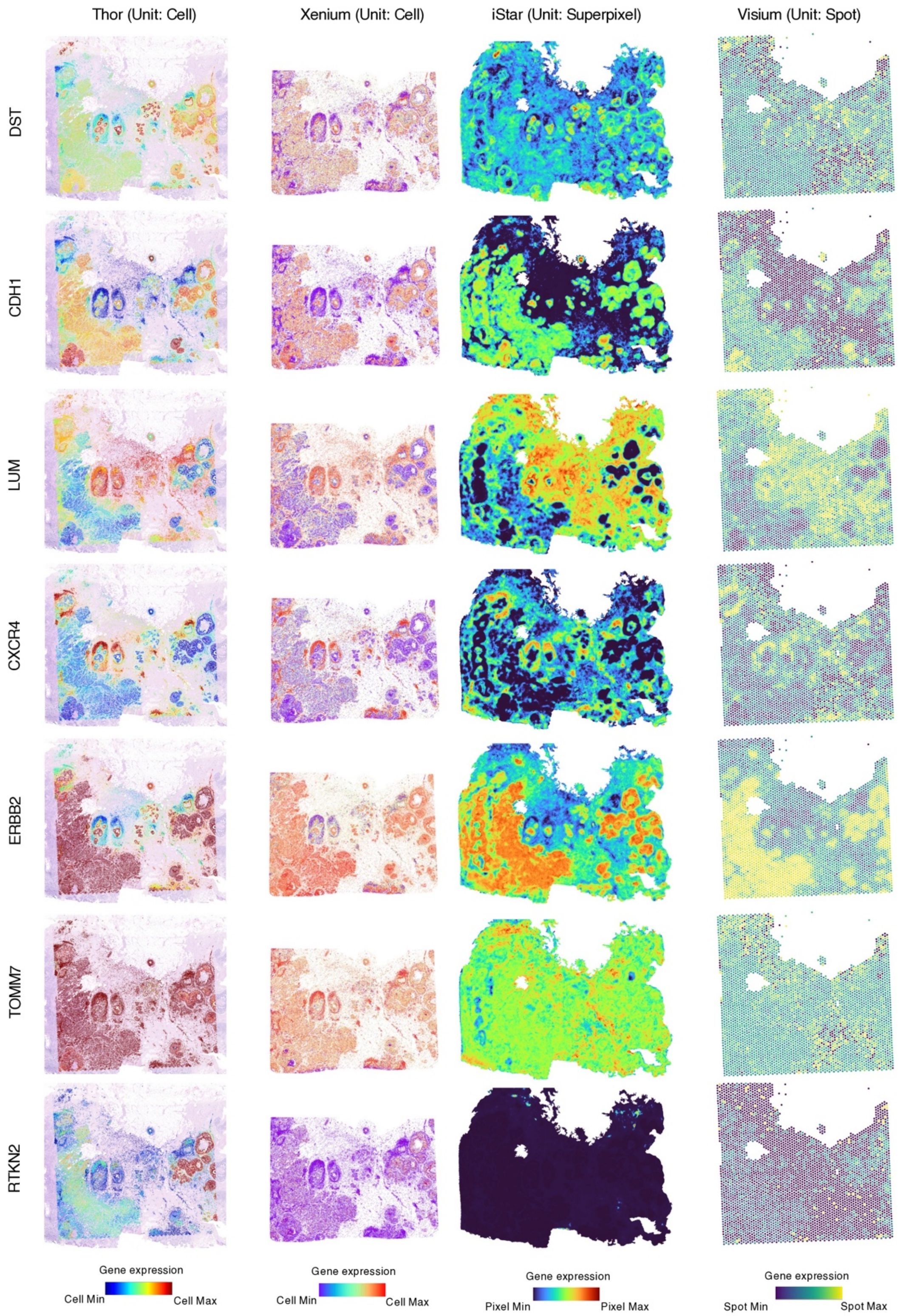

Supplementary Figure 8: Expression profiles of genes in the human breast cancer tissues. Data predicted by Thor and iStar, along with Xenium and Visium measurement are compared**.** For visualization, gene expression levels predicted by Thor and iStar are normalized in the whole tissue.

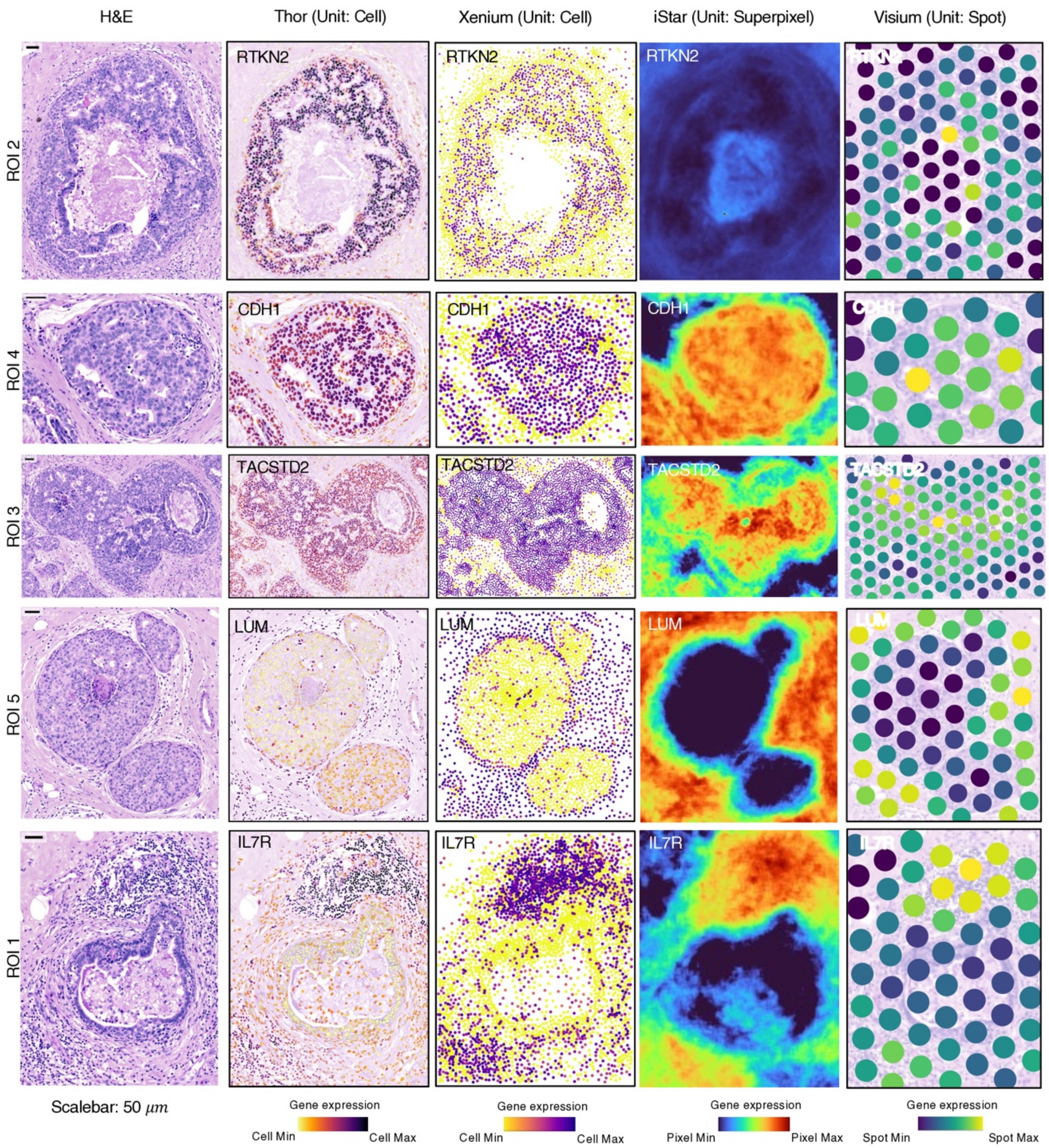

Supplementary Figure 9: Expression profiles of genes in ROIs of the human breast cancer tissues. Data predicted by Thor and iStar, along with measured by Xenium and Visium are compared in ROIs. H&E images of the ROIs are provided for reference. For visualization, gene expression levels predicted by Thor and iStar are normalized in the ROI.

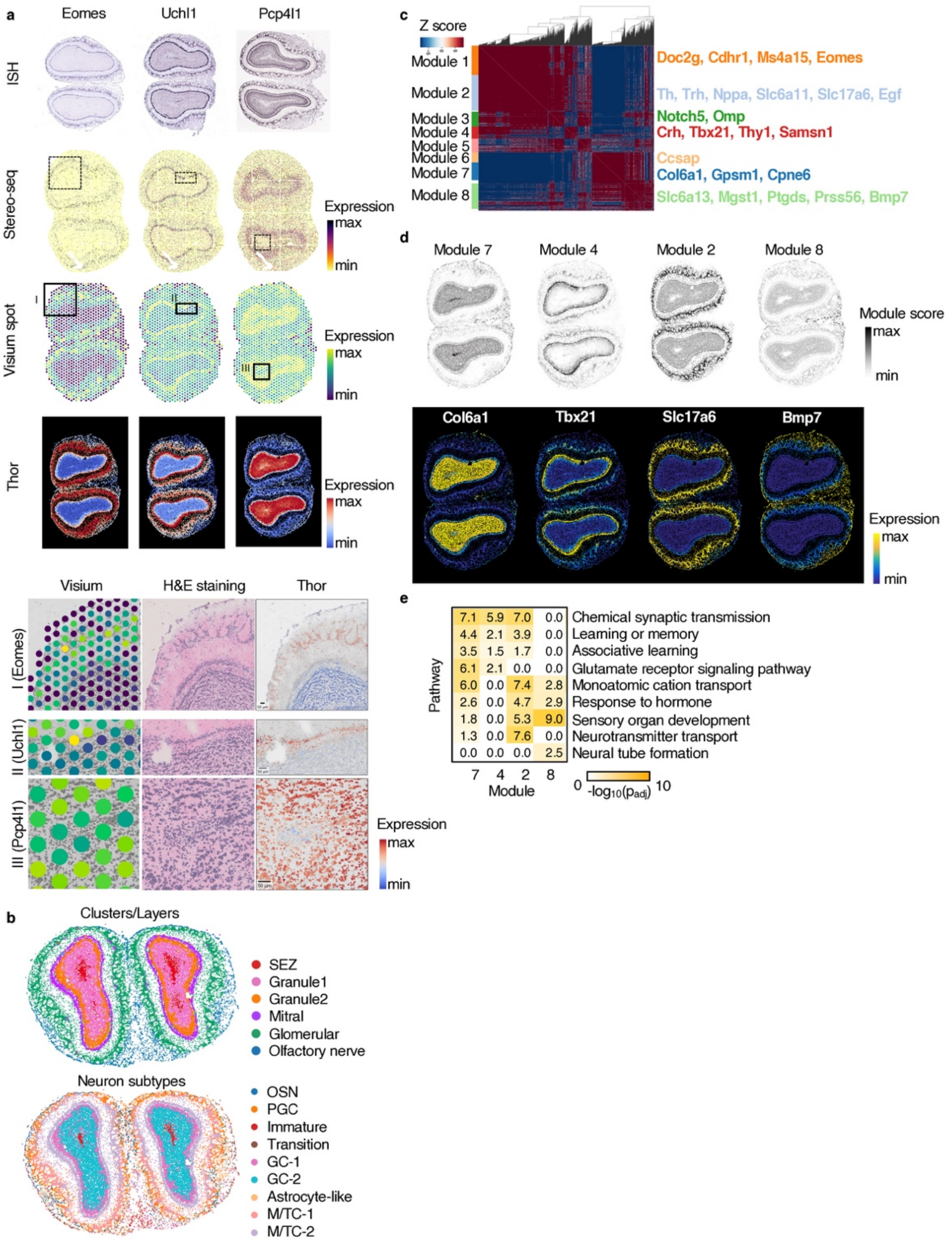
a
Eomes
Uchl1
Pcp4l1
ISH
Stereo-seq
Visium spot
Thor
Expression
max
min
Visium
H&E staining
Thor
I (Eomes)
II (Uchl1)
III (Pcp4l1)
b
Clusters/Layers
SEZ
Granule1
Granule2
Mitral
Glomerular
Olfactory nerve
Neuron subtypes
OSN
PGC
Immature
Transition
GC-1
GC-2
Astrocyte-like
M/TC-1
M/TC-2
c
Z score
Module 1
Module 2
Module 3
Module 4
Module 5
Module 6
Module 7
Module 8
Doc2g, Cdhr1, Ms4a15, Eomes
Th, Trh, Nppa, Slc6a11, Slc17a6, Egf
Notch5, Omp
Crh, Tbx21, Thy1, Samsn1
Ccsap
Col6a1, Gpsm1, Cpne6
Slc6a13, Mgst1, Ptgds, Prss56, Bmp7
d
Module 7
Module 4
Module 2
Module 8
Module score
max
min
Col6a1
Tbx21
Slc17a6
Bmp7
Expression
max
min
e
Pathway
7.1 5.9 7.0 0.0 Chemical synaptic transmission
4.4 2.1 3.9 0.0 Learning or memory
3.5 1.5 1.7 0.0 Associative learning
6.1 2.1 0.0 0.0 Glutamate receptor signaling pathway
6.0 0.0 7.4 2.8 Monoatomic cation transport
2.6 0.0 4.7 2.9 Response to hormone
1.8 0.0 5.3 9.0 Sensory organ development
1.3 0.0 7.6 0.0 Neurotransmitter transport
0.0 0.0 0.0 2.5 Neural tube formation
7 4 2 8
Module
0 -log10(padj) 10

Supplementary Figure 10: Thor reveals detailed mouse olfactory bulb layers. (a) Spatial expression profiles of genes in the mouse olfactory bulb using the ISH (first row), Stereo-seq (second row), Visium (third row), and Thor-inferred (fourth row) data. Selected regions (black boxes) are zoomed in for detailed inspection of gene expression in glomerular, mitral, and SEZ layers. (b) Spatial distribution of cell clusters and neuron subtypes based on the Thor-inferred single-cell gene expression. OSN: Olfactory sensory neuron, PGC: Periglomerular cell, GC: Granule cell, M/TC: Mitral/tufted cell. (c) Genes are grouped into 8 modules based on pairwise local correlation using the package Hotspot. Marker genes for MOB layers are shown along corresponding gene modules. (d) Module scores of four gene modules are visualized with spatial context, as well as the Thor-inferred single-cell expression profiles of representative genes in the four modules. (e) GO pathway enrichment of the four representative gene modules. Source data are provided in a Source Data file.

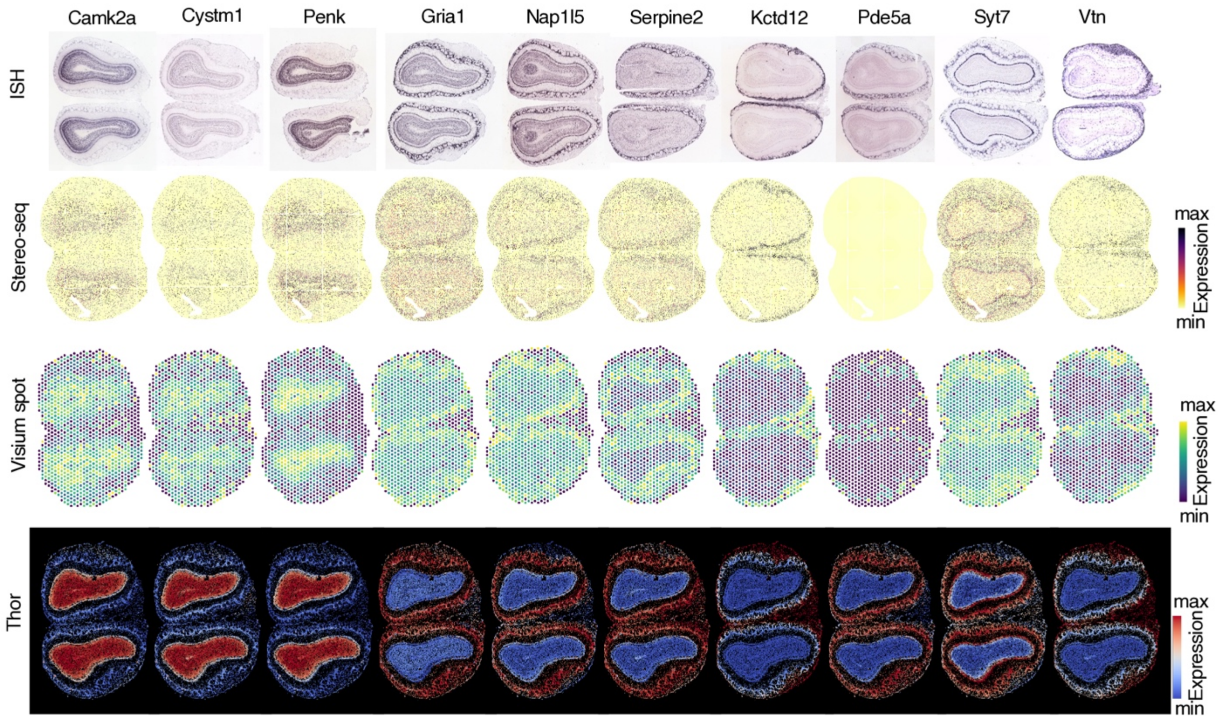

Supplementary Figure 11: Comparison between predicted expression profiles and gene expression patterns measured from other sources in MOB. The top row is the ISH images downloaded from Allen brain atlas; the second row is the predicted gene expression using Thor; the third row is the gene expression measured by Stereo-seq; the fourth row is the spot-level gene expression sequenced by Visium from 10x Genomics.

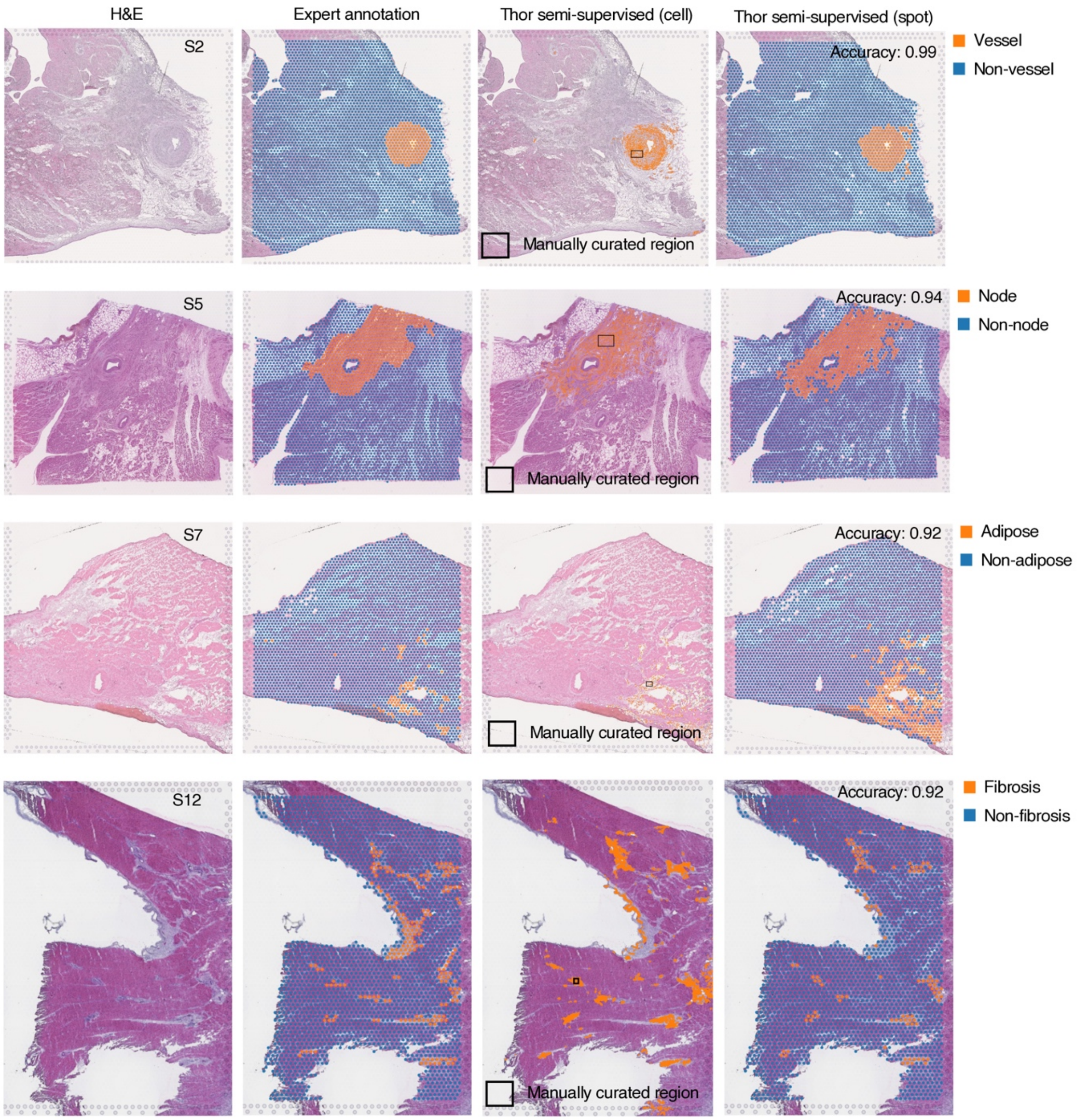

Vessel			Node			Adipose			Fibrosis		
	Accuracy	AUC		Accuracy	AUC		Accuracy	AUC		Accuracy	AUC
S1	0.99	0.88	S4	0.98	0.87	S7	0.92	0.89	S10	0.93	0.72
S2	0.99	0.95	S5	0.94	0.87	S8	0.85	0.62	S11	0.93	0.64
S3	0.94	0.73	S6	0.92	0.76	S9	0.84	0.76	S12	0.92	0.65

Supplementary Figure 12: Quantitative assessment of SSA against spot-level expert annotations. (left panel) H&E image, (second panel) spot-level pathology annotations, (third panel) Thor's cell-level annotations, and (right panel) Thor's cell-level annotation mapped to the spots using majority voting (>50%) of representative samples in each tissue type including vessel, nodal tissue, adipose tissue, and fibrosis. The metrics are calculated based on spot-level annotations.

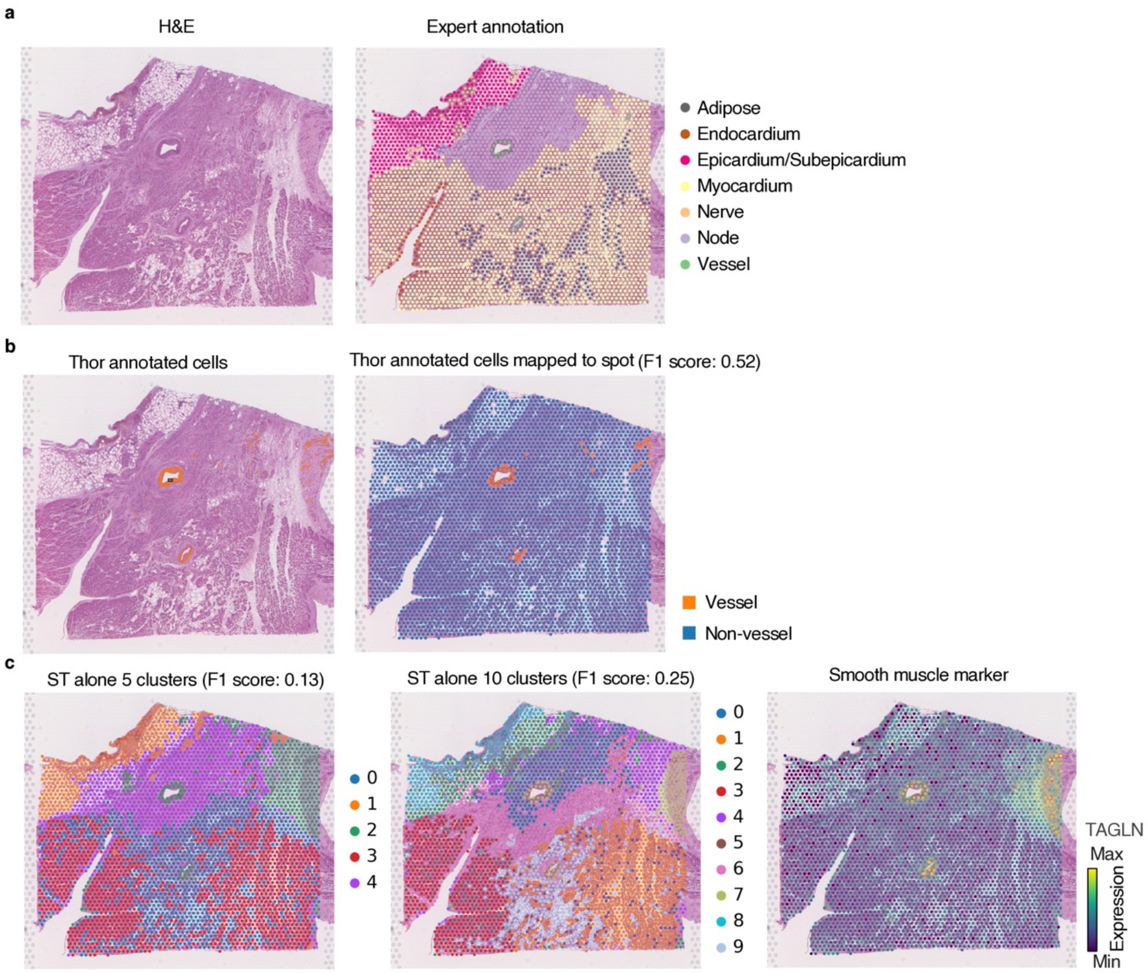

Supplementary Figure 13: Comparison between SSA and the spot-level clustering. (a) H&E staining image and expert annotations on the heart tissue sample. (b) Cells annotated via SSA. Annotation of cells are mapped to corresponding spots according to majority voting for quantitative evaluation against expert annotations. (c) k-means clusters solely based on Visium ST data. Regardless of the number of clusters used, Visium ST data alone fails to accurately distinguish vessel-associated spots (enriched with smooth muscle and endothelial cells) from some myocardium spots with high *TAGLN* (a smooth muscle marker) expression.

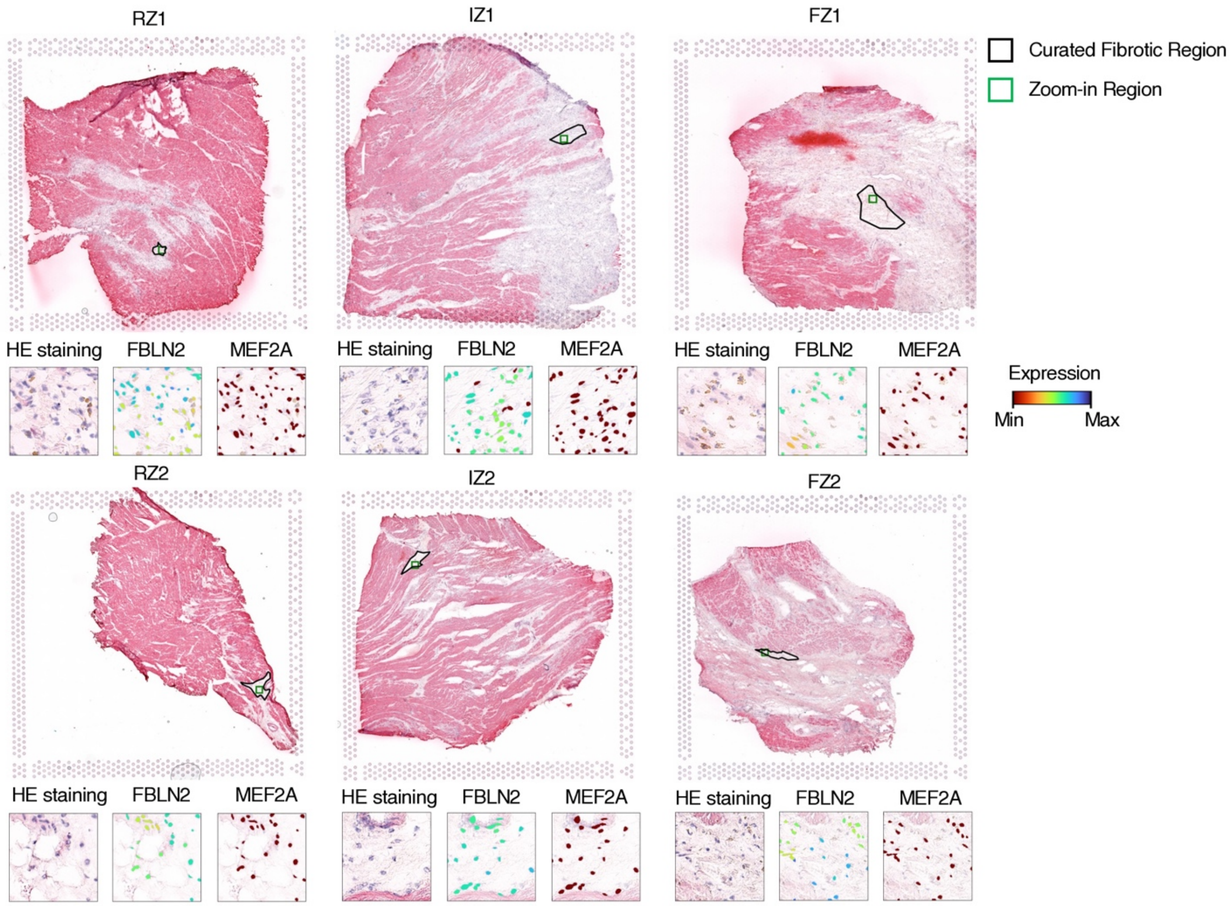

Supplementary Figure 14: Curated fibrotic ROIs in human myocardial infarction tissues. H&E staining images of all the tissues from remote, ischaemic, and fibrotic zones. The predicted single-cell expression of the fibroblast marker gene (*FBLN2)* and cardiac muscle-associated gene (*MEF2A)* are visualized in the curated ROIs.

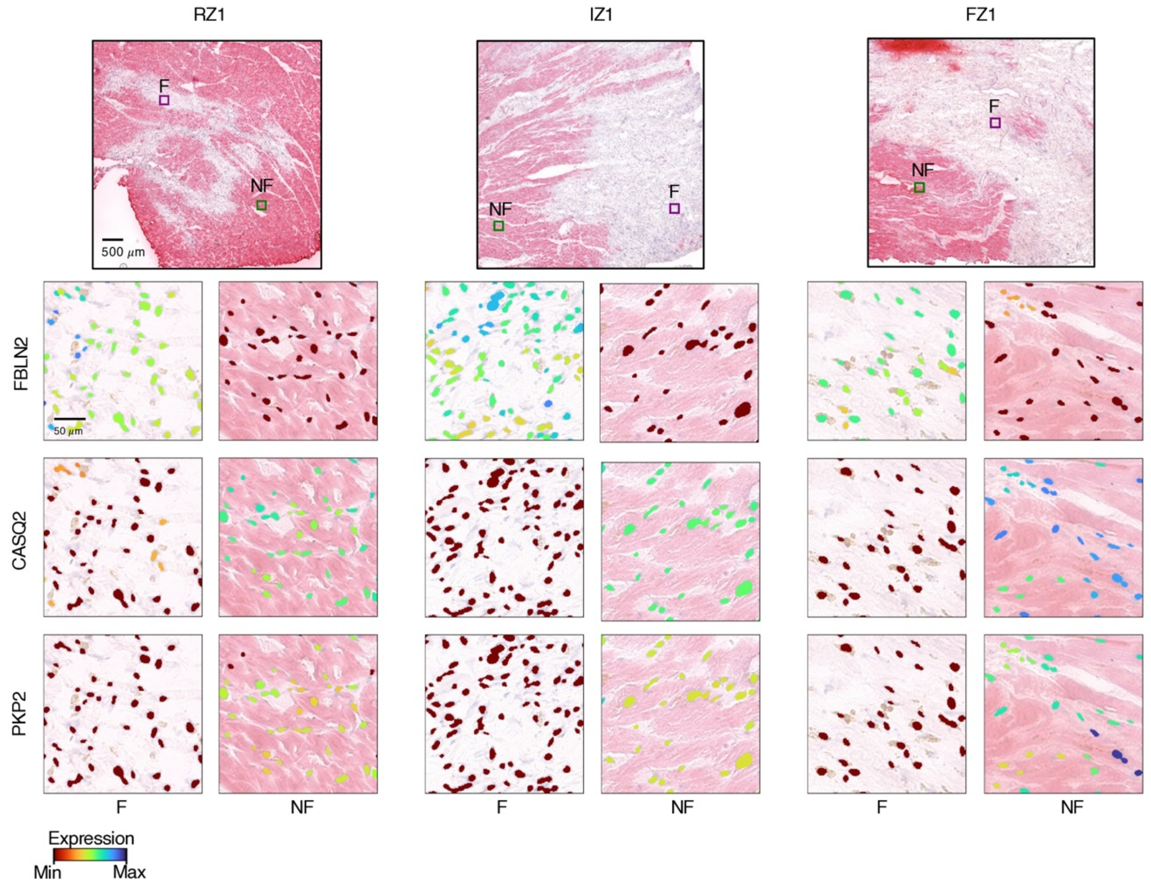

Supplementary Figure 15: DEGs between the curated fibrotic and non-fibrotic regions in the RZ1, IZ1, and FZ1 tissues. H&E staining images of RZ1, IZ1, and FZ1 tissues and Thor-predicted single-cell expression of the fibroblast marker gene (*FBLN2)* and cardiac muscle-associated genes (*CASQ2* and *PKP2)* are visualized in the curated ROIs. F and NF stand for fibrotic region and non-fibrotic region, respectively.

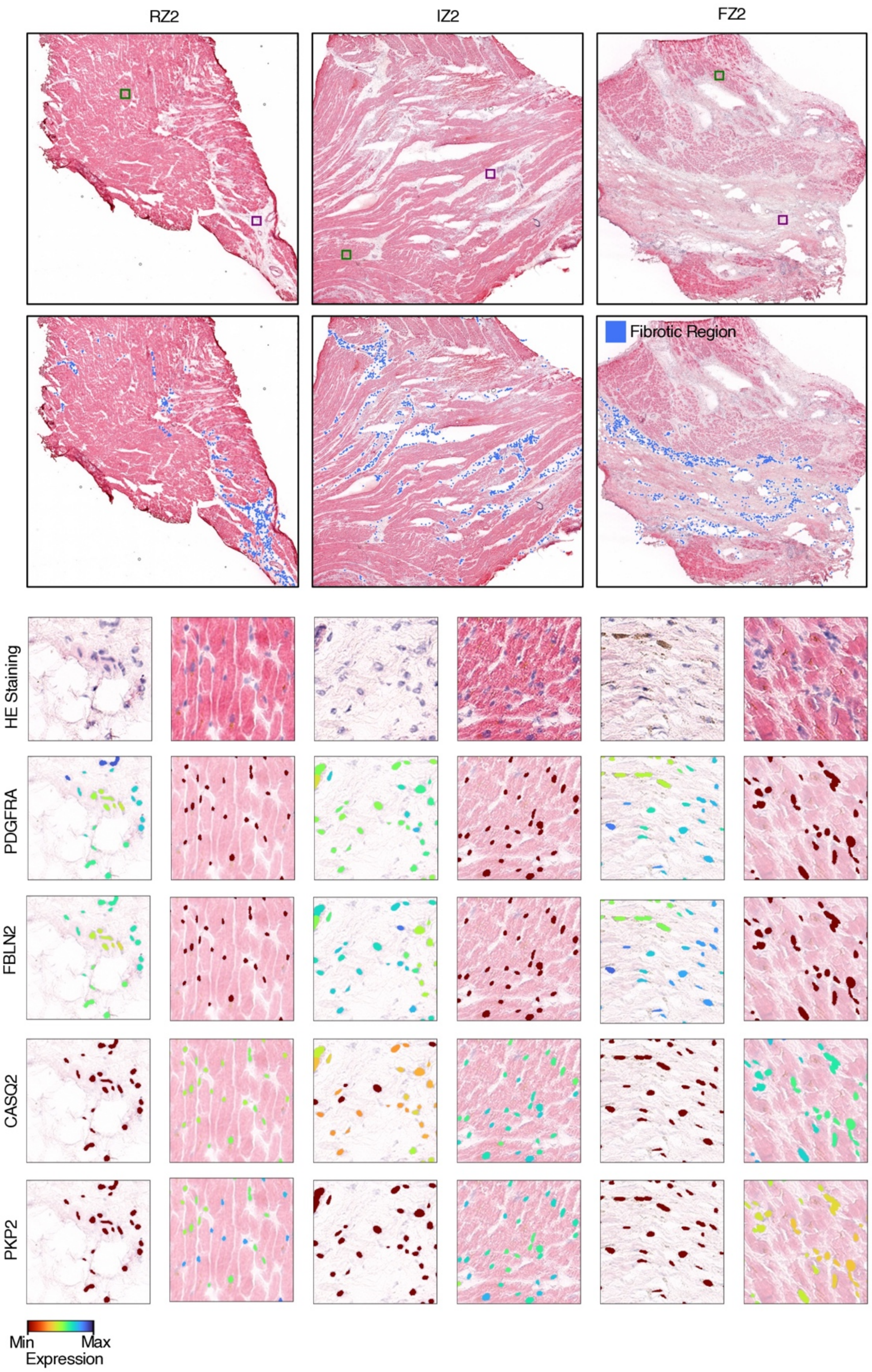

Supplementary Figure 16: SSA selects fibrotic regions in shallow areas in human myocardial infarction tissues. Thor-annotated fibrotic regions (blue) are visualized on the H&E staining images of RZ2, IZ2, and FZ2 tissues. Thor-predicted cell-level expression profiles of fibroblast marker genes (*PDGFRA* and *FBLN2)* and cardiac muscle-associated genes (*CASQ2* and *PKP2)* are visualized in the curated ROIs.

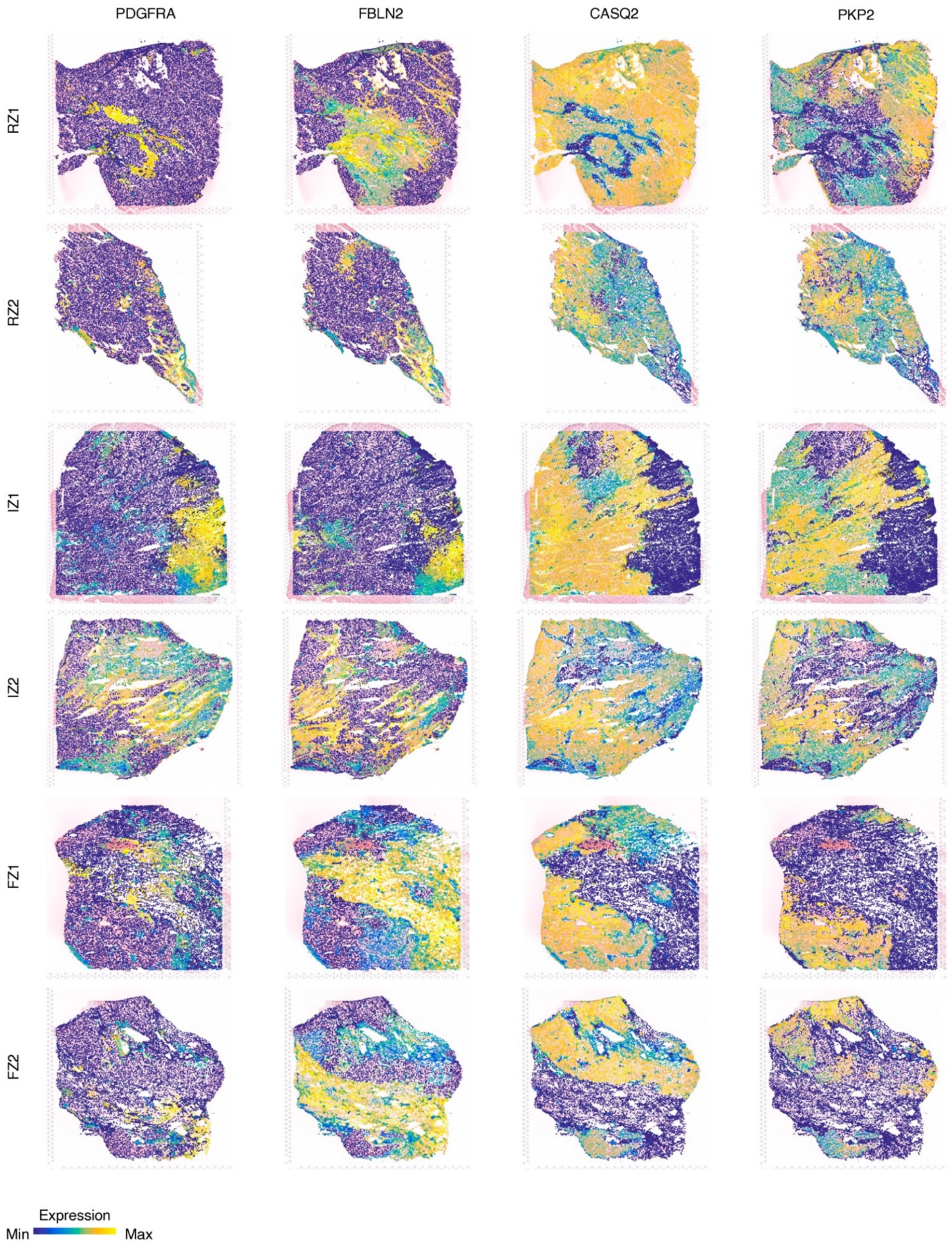

Supplementary Figure 17: Expression patterns of fibrotic-specific and cardiac muscle-associated genes in human myocardial infarction tissues. Fibroblast marker genes (*PDGFRA* and *FBLN2),* and cardiac muscle-associated genes (*CASQ2* and *PKP2)* are visualized on top of the whole tissues.

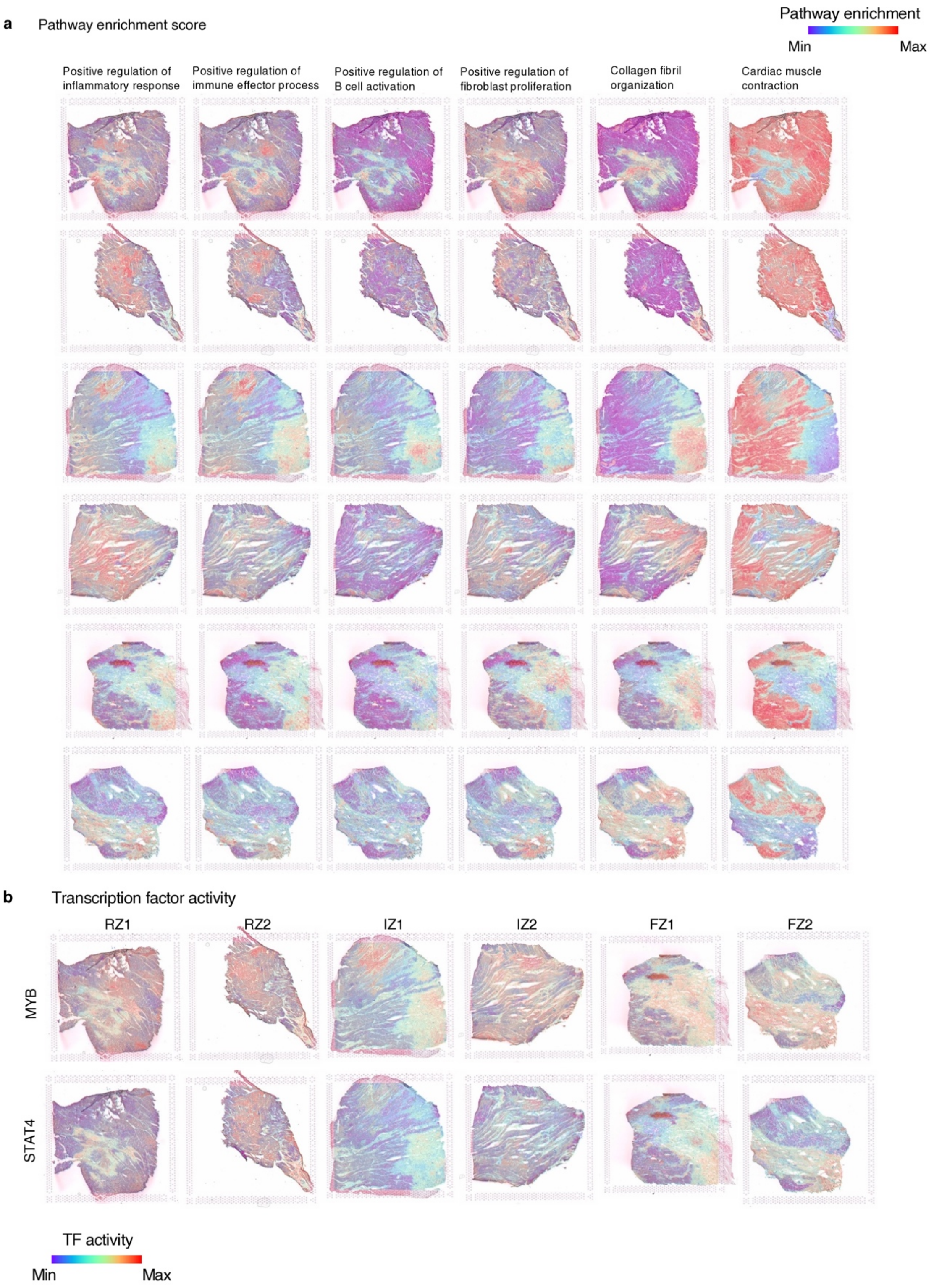

Supplementary Figure 18: GO pathway enrichment and TF activity in human heart tissues with MI. (a) The GO pathway enrichment scores are calculated based on the gene expression in each *in silico* cell using the Python library *decoupler*. (b) The TF activity scores are calculated based on the gene expression in each *in silico* cell. The database CollecTRI and Python library *decoupler* are used for inferring the TF activity.

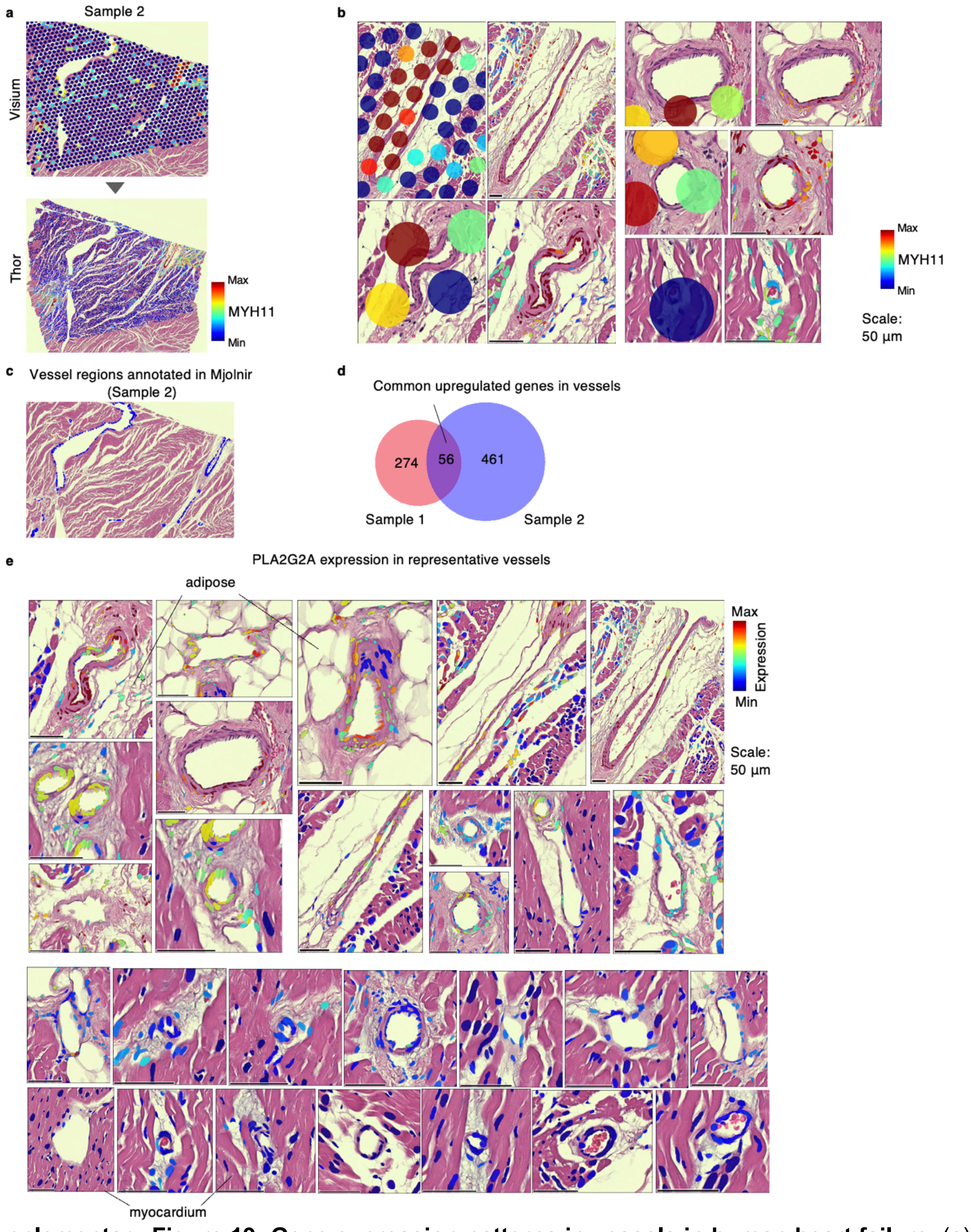

Supplementary Figure 19: Gene expression patterns in vessels in human heart failure. (a) *MYH11* expression at the spot level and the cell level across the whole tissue 'sample 2'. (b) *MYH11* expression at the spot resolution and the cell resolution in representative vessels. (c) Vessel regions in 'sample 2'. (d) Venn diagram of the upregulated genes in the vessels across two samples. (e) *PLA2G2A* expression in representative vessels from post-LVAD patient tissues.

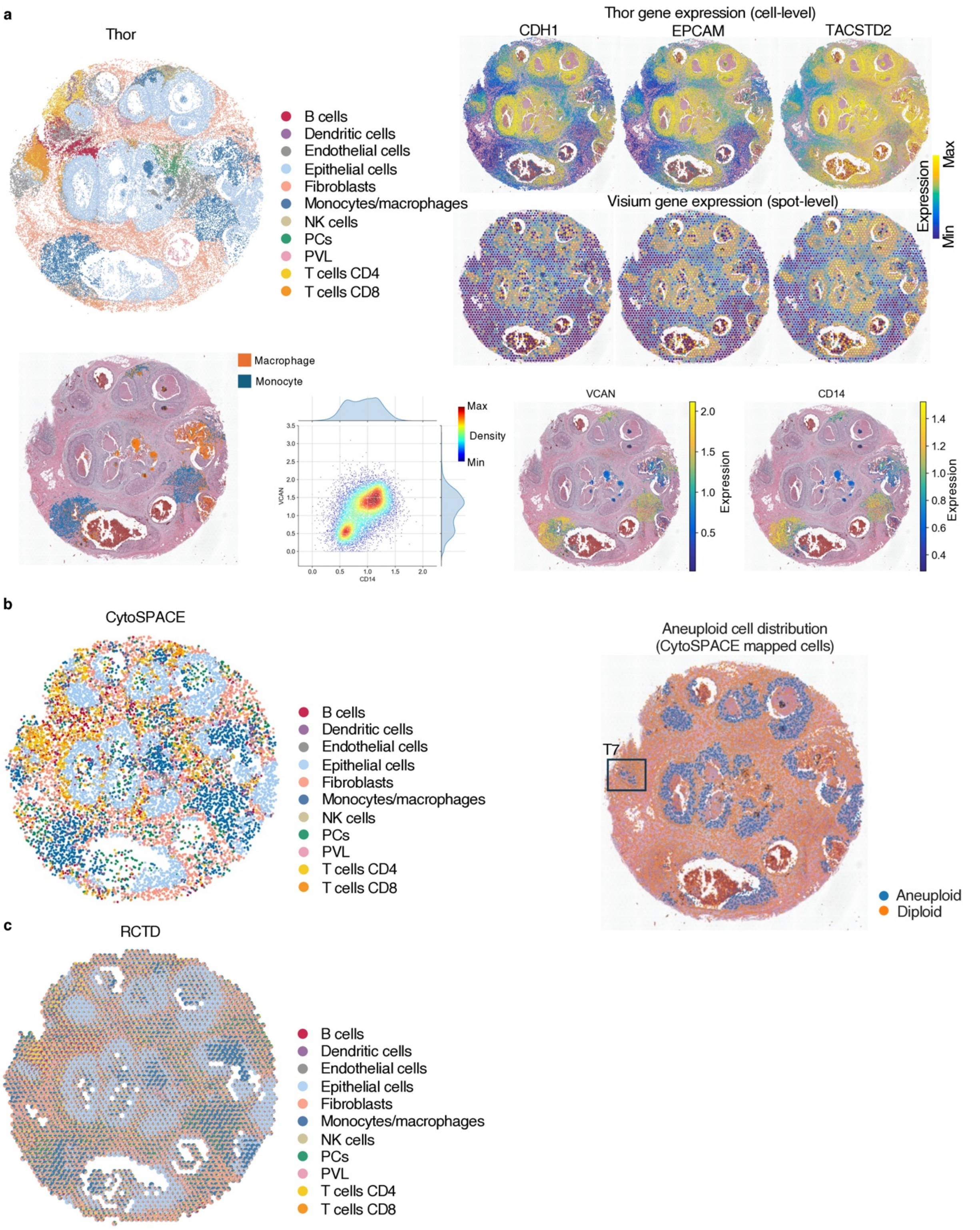

Supplementary Figure 20: The inferred cell type distribution in DCIS by (a) Thor, (b) CytoSPACE, and (c) RCTD. Expression profiles of tumor and macrophage marker genes are provided for reference. The aneuploid (tumor) and diploid (non-tumor) cell distributions inferred by CopyKAT using CytoSPACE-mapped gene expression is comparable to Thor result.

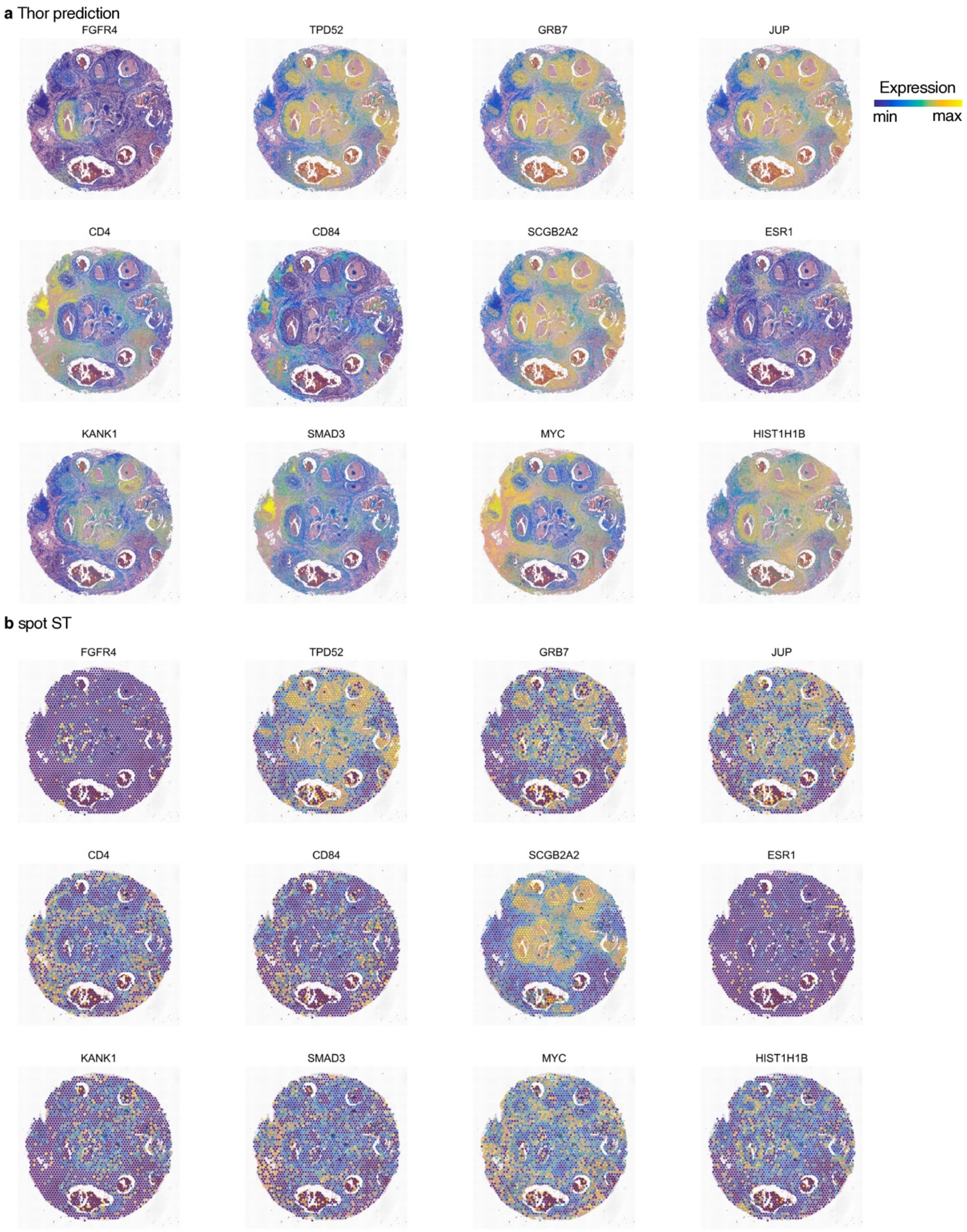

Supplementary Figure 21: Expression profiles of genes in DCIS. (a) Thor-predicted single-cell spatial gene expression; (b) Spot-resolution spatial gene expression from the Visium data.

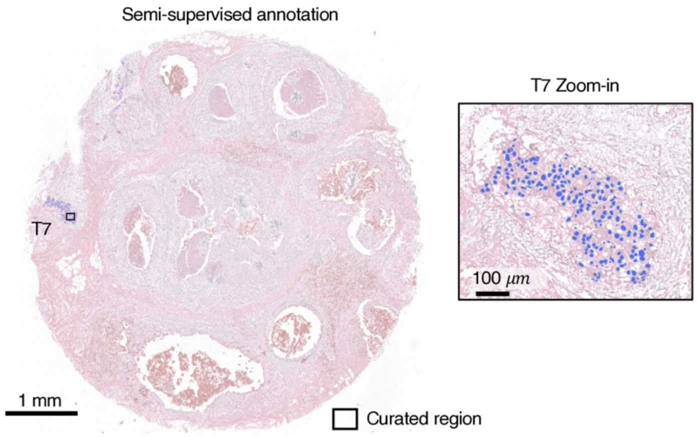

Supplementary Figure 22: SSA selects tumor cells in tumor region T7 in DCIS. The curated rectangular region is marked with a black box.

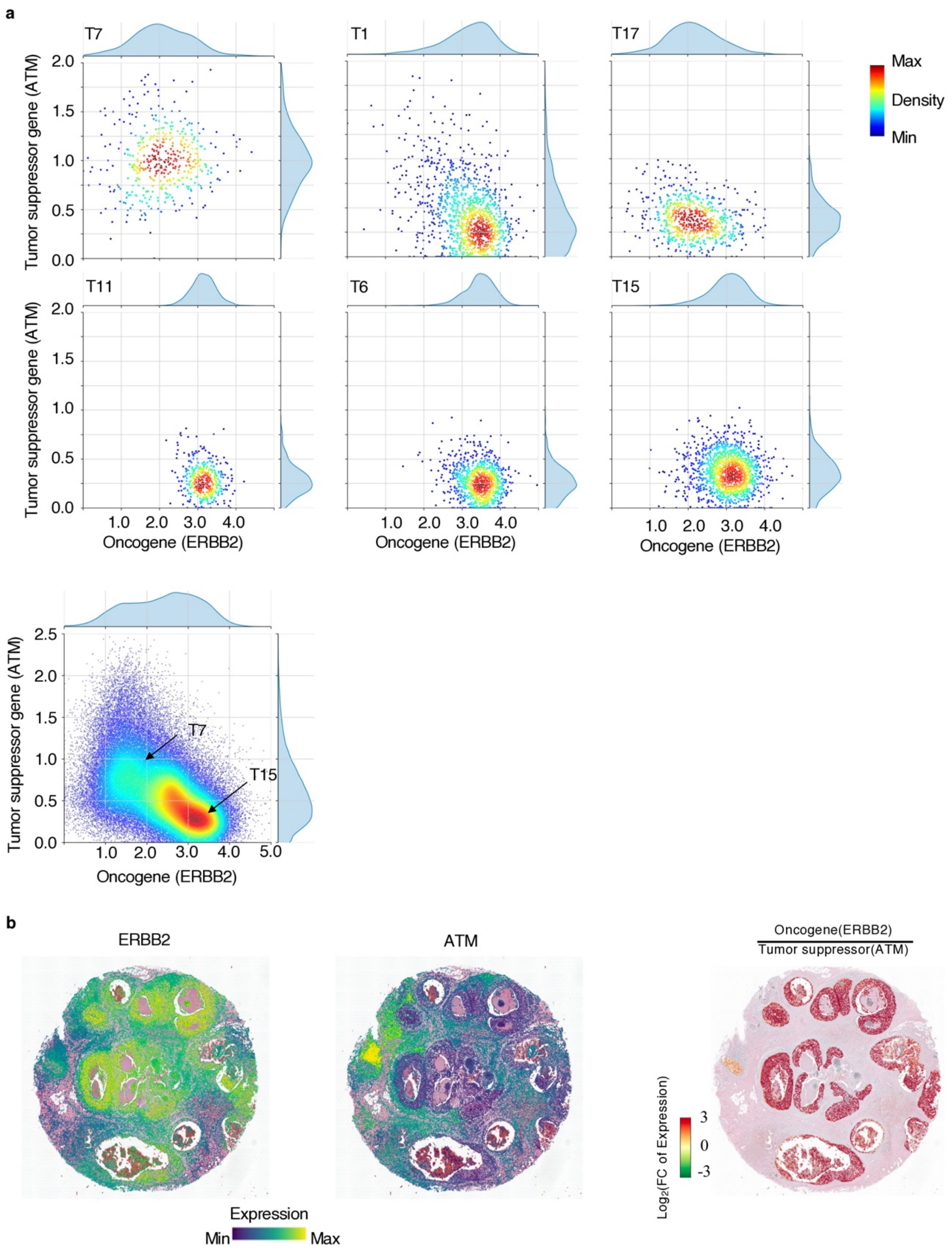

Supplementary Figure 23: 2D density plots of the expression of oncogene and tumor suppressor gene in DCIS. (a) Expressions of the oncogene *ERBB2* and tumor suppressor gene *ATM* are plotted in tumor regions with highest TLS scores (T7, T1, and T14) and lowest TLS scores (T11, T6, and T15), where cells are colored according to the density. (b) The predicted spatial expression levels of the two genes by Thor and the fold changes between the oncogene and the tumor suppressor.

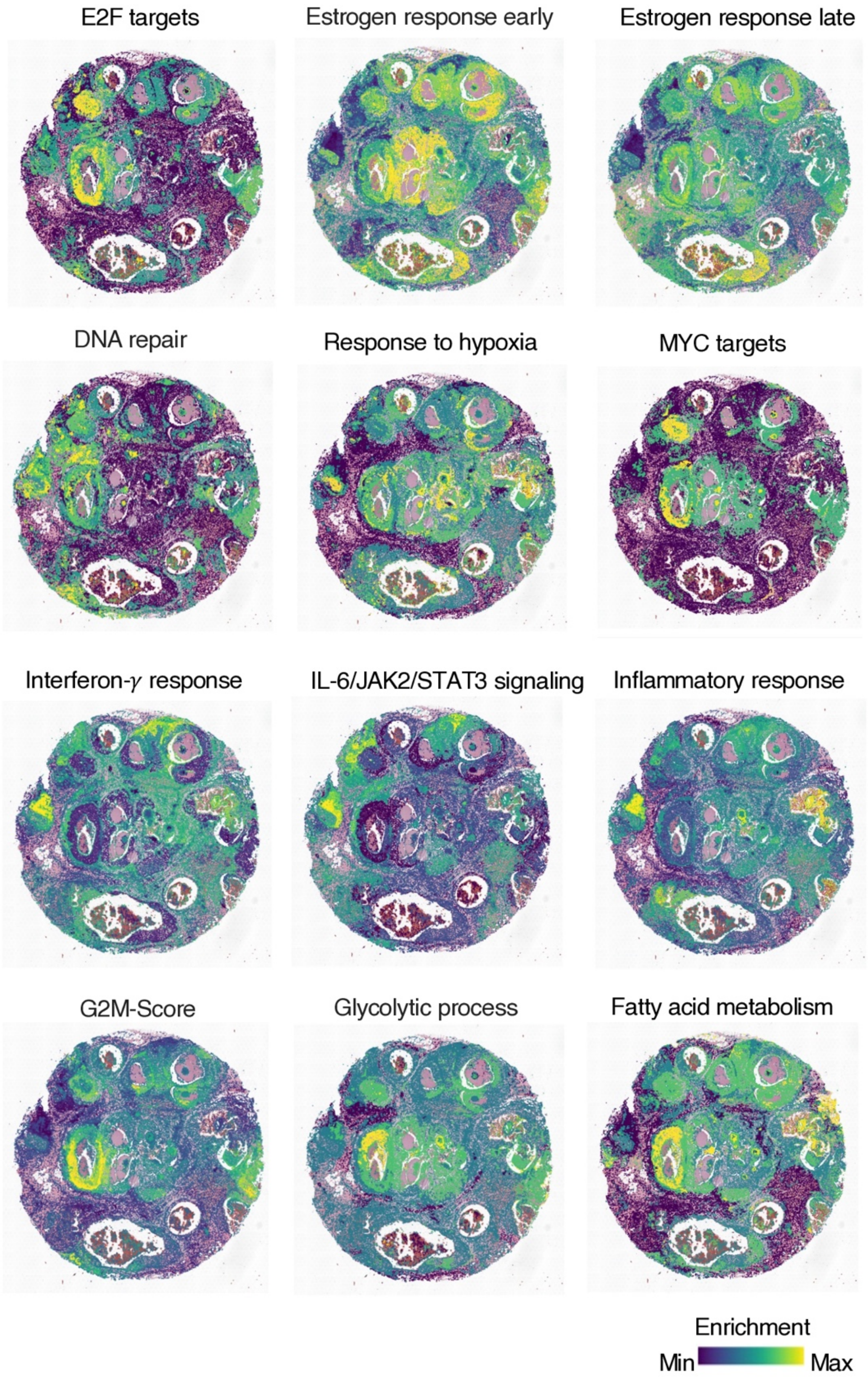

Supplementary Figure 24: Enrichment of hallmark pathways in DCIS. The hallmark pathway enrichment is performed using *decoupler* with Thor-predicted cell-level transcriptome and the MSigDB hallmark gene sets as input.

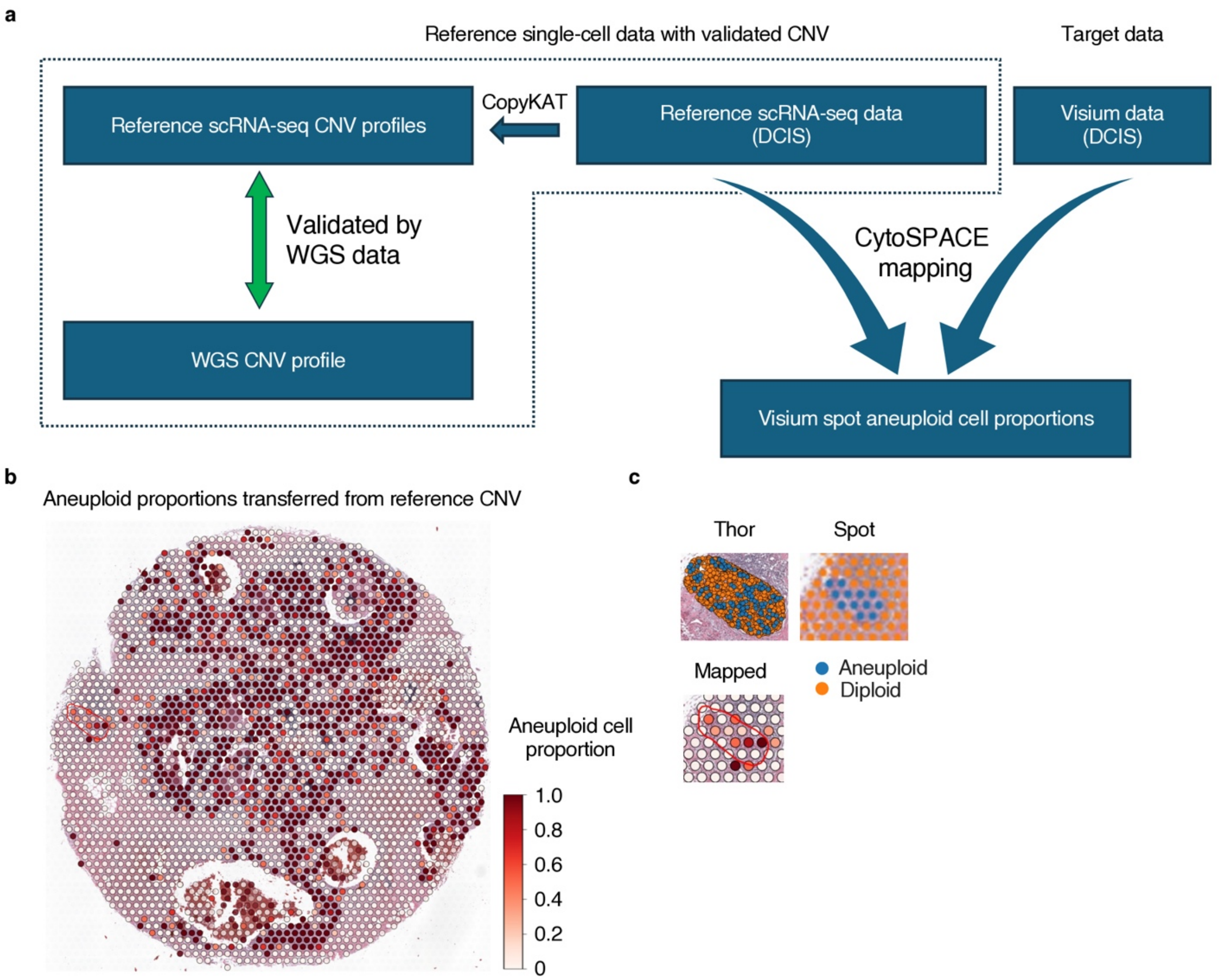

Supplementary Figure 25: Validation of CNV profiles. (a) Designed approach for obtaining WGS-validated Visium CNV profiles. CytoSPACE was utilized to transfer CNV profiles of single cells, which were validated by WGS DNA-seq data, to Visium spots. (b) Spot-level aneuploid proportions mapped by CytoSPACE. (c) Aneuploid distribution in tumor region T7 predicted by Thor, raw Visium, and mapped by CytoSPACE (reference).

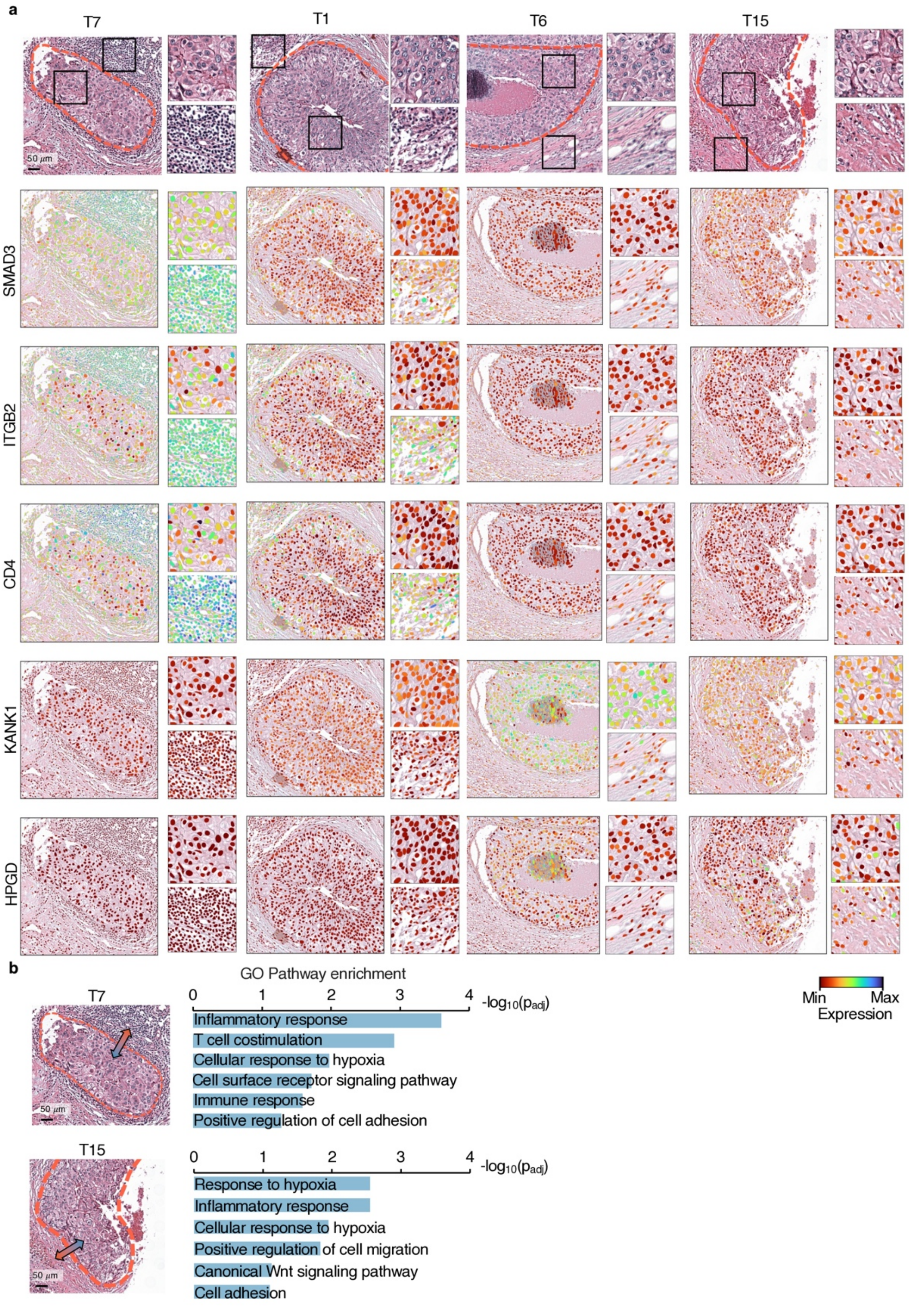
a
T7
T1
T6
T15
50 μm
SMAD3
ITGB2
CD4
KANK1
HPGD
b
GO Pathway enrichment
T7
0
1
2
3
4
-log10(padj)
Inflammatory response
T cell costimulation
Cellular response to hypoxia
Cell surface receptor signaling pathway
Immune response
Positive regulation of cell adhesion
50 μm
T15
0
1
2
3
4
-log10(padj)
Response to hypoxia
Inflammatory response
Cellular response to hypoxia
Positive regulation of cell migration
Canonical Wnt signaling pathway
Cell adhesion
50 μm
Min
Max
Expression

Supplementary Figure 26: Comparison of tumor regions with highest and lowest TLS scores and their interactions with surrounding environments. (a) Zoom-in view of the tumor regions of highest (T7 and T1) and lowest (T6 and T15) TLS scores. Thor-predicted expression of DEGs is visualized in the inner and perimetral parts of the tumor regions. (b) Interaction between tumor regions and the surrounding environments. The orange dashed lines mark the pathology-annotated tumor region boundaries. Source data are provided in a Source Data file.

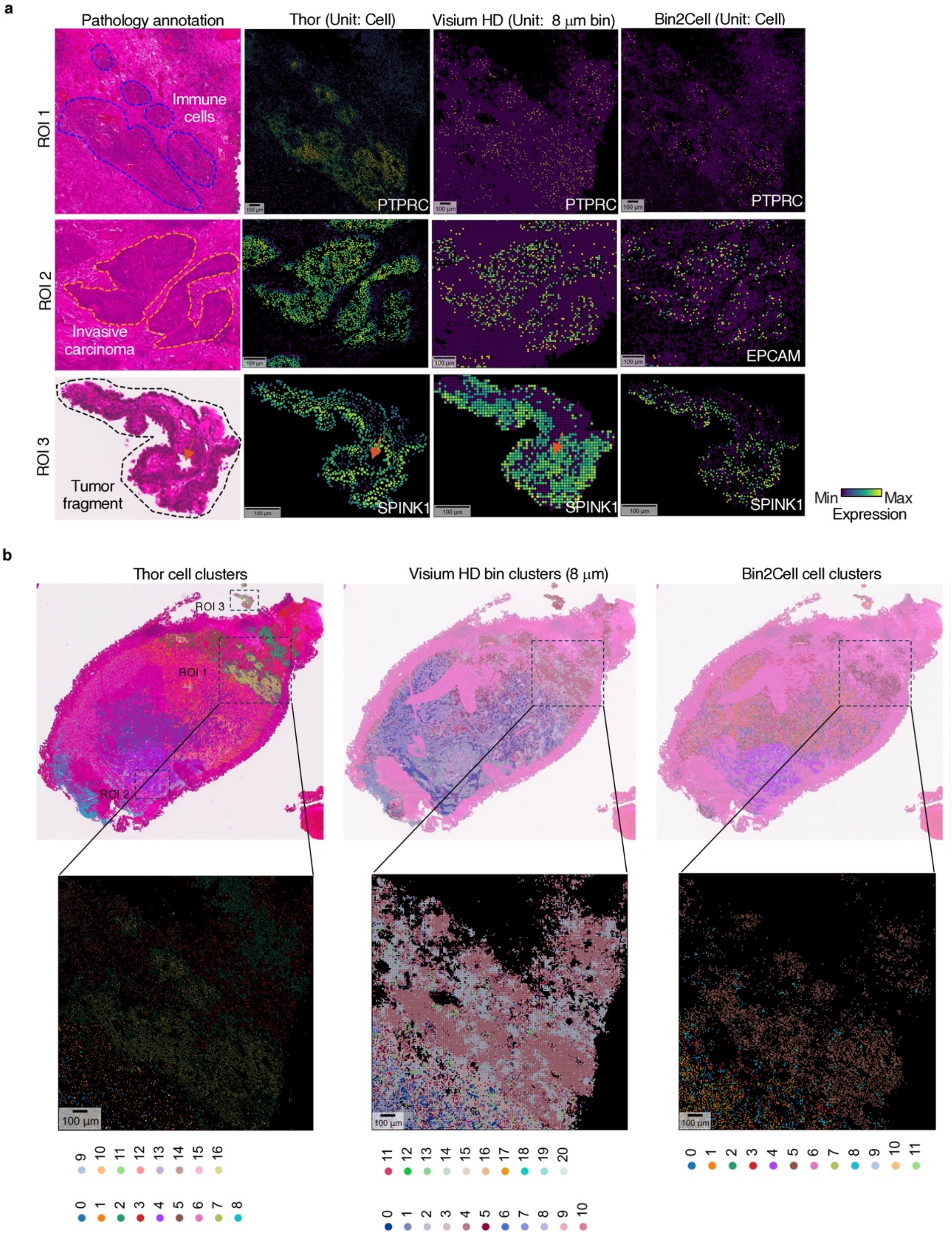

Supplementary Figure 27: Thor imputes Visium HD data and reconstructs gene expression patterns that align with pathology annotations in a bladder cancer sample. (a) Pathology annotations (left) highlight immune cells (ROI 1), invasive carcinoma (ROI 2), and a tumor fragment (ROI 3). Gene expression patterns inferred by Thor (middle), from Visium HD (right), and inferred by Bin2Cell (right). The orange arrow points to a region with no cell. (b) Cell clusters with a zoomed-in view of ROI 1 by Thor (left), bin clusters by Visium HD 8 μm bin data (middle) and cell clusters by Bin2Cell (right; with empty cells removed).

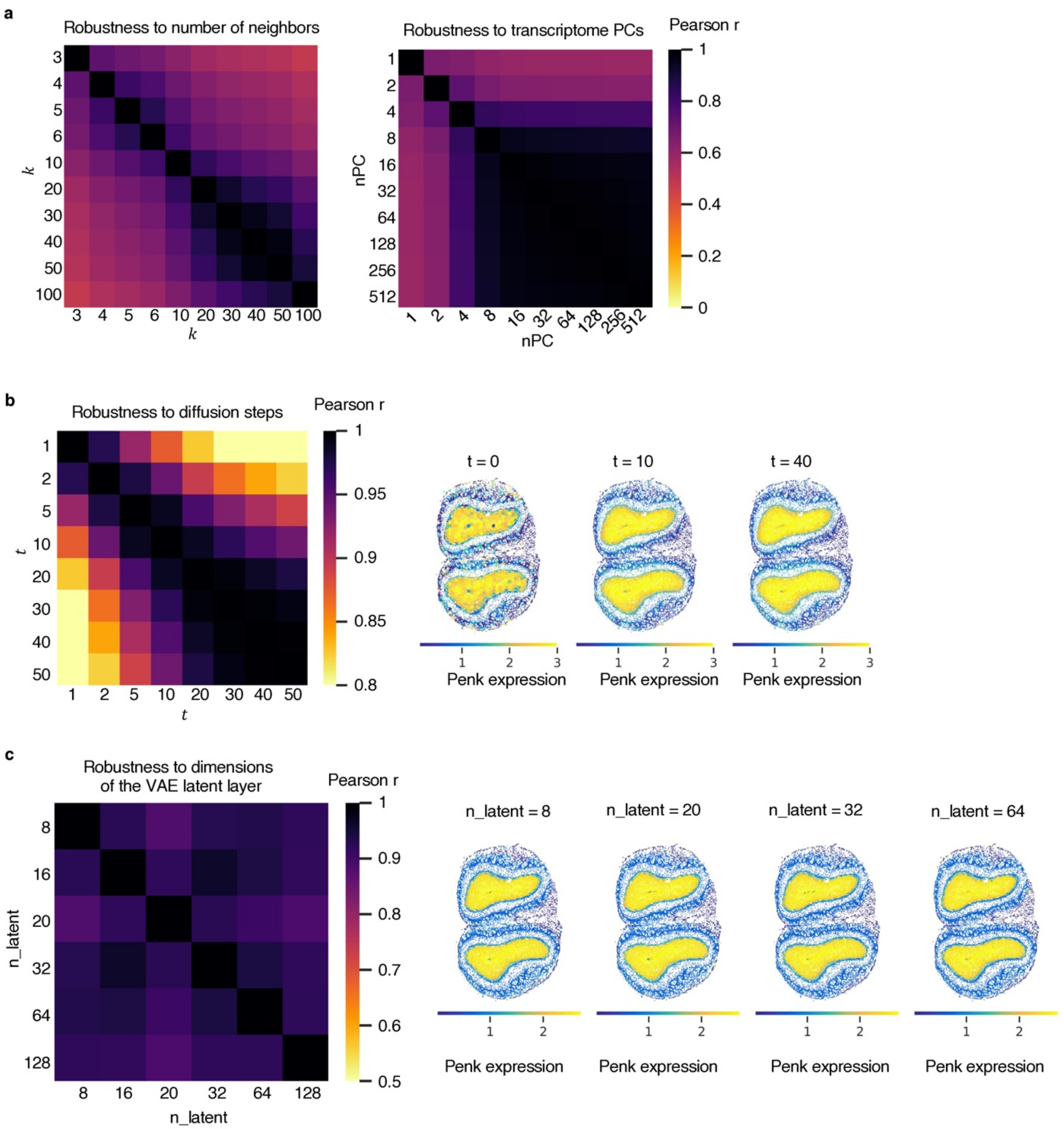

Supplementary Figure 28: Sensitivity analyses of Thor inference on (a) graph construction parameters, (b) the diffusion steps, and (c) VAE latent dimensions. Mean Pearson correlation coefficients between every pair of parameter setttings for all genes are plotted. Spatial distributions of a representative gene *Penk* are provided to illustrate the influence of diffusion steps and latent dimension on infererence.

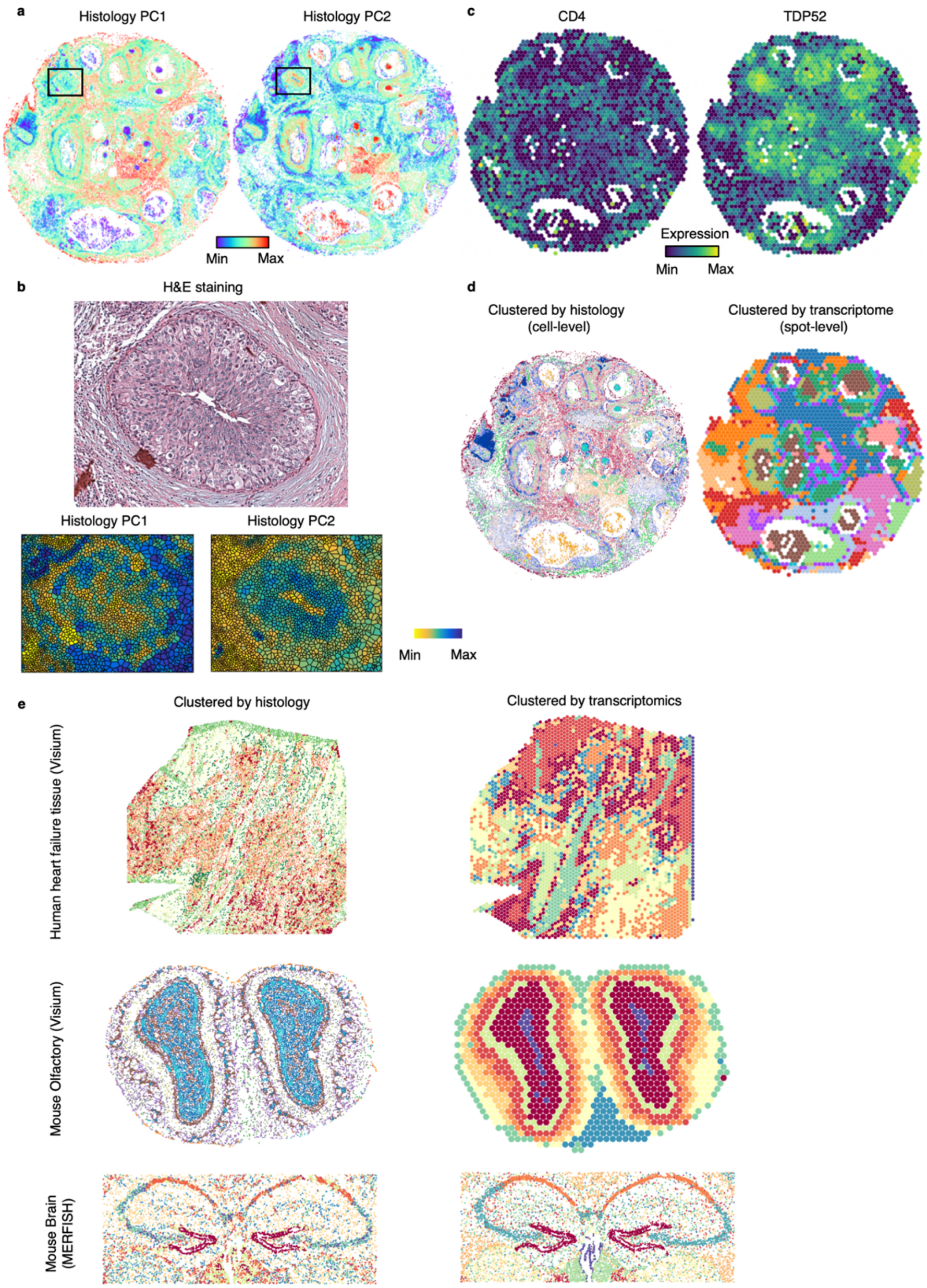

Supplementary Figure 29. Histology and spatial transcritomics reveal tissue structure in respected resolutions. (a) Projection of image features to the first two principal components (PCs) in the DCIS tissue image. The black boxes mark the region of interest (ROI). **(b)** Projection of histological features to the first two PCs in a tumor region in DCIS tissue. **(c)** Spot-level gene expression of *CD4* and *TDP52* from the DCIS Visium data. **(d)** Histology feature clusters and transcriptome clusters of DCIS Visium data. The cells or spots are colored according to the clusters. **(e)** Histology feature clusters and transcriptome clusters of human heart failure tissue (Visium data), mouse olfactory bulb (Visium data) and mouse brain cerebellum (MERFISH). The cells or spots are colored according to the clusters.

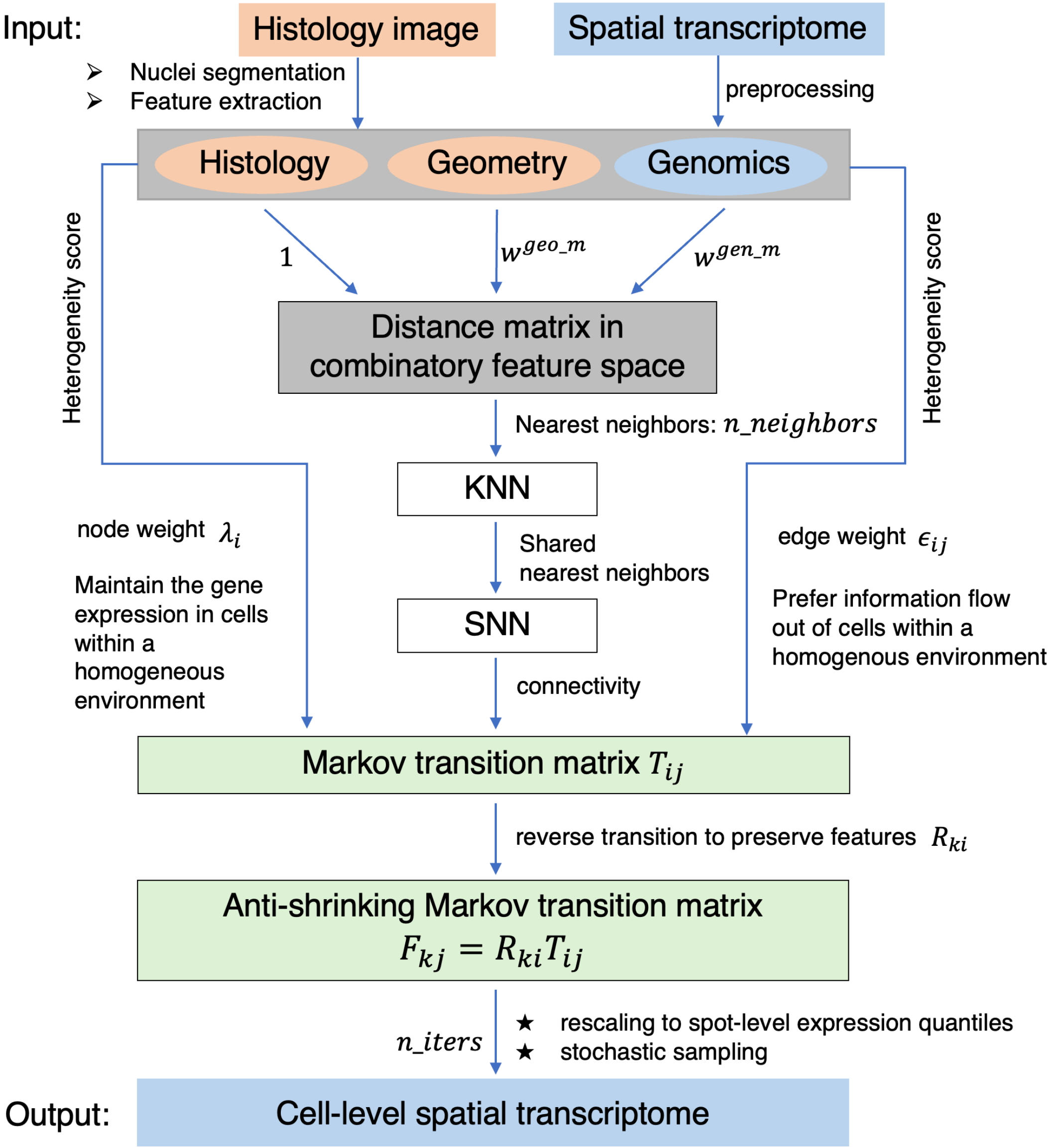

Supplementary Figure 30. Workflow of Thor inference. First, extract image features from the histology image, including locations of *in silico* cells and contextual features of each cell in their spatial neighborhood. Meanwhile, the spatial transcriptome data is preprocessed and the gene expression on the segmented cells is initialized according to the nearest spot. Second, compute multi-modal distances between cells and construct the cell-cell network SNN based on their morphological features, geometrical locations, and the reduced-dimension transcriptome weighted by user inputs. Third, convert the distances into affinities using a Gaussian kernel, so that similarity between two cells decreases exponentially with their distance. Fourth, construct a Markov transition matrix for transitioning information between similar cells and prohibiting information flow from cells within a heterogenous environment. A reverse diffusion transition matrix is constructed to prevent the shrinking of the data space. Fifth, the cell-level spatial transcriptome is inferred by multi-step diffusion with the constructed transition matrix.

Supplementary Note 1

There is an inherent structure in the cells in a tissue probed by different sources of measurements, such as imaging and genomic sequencing. Spatial transcriptomics is one of the most pursued technologies as it reveals the physical tissue structure and molecular characteristics simultaneously. There are two sources of complementary information: the whole slide image (WSI) is of high subcellular spatial resolution but of low dimension, while the spatial transcriptome (ST) is of high dimension but lower spatial resolution (spot level). In this note, we show the two sources of information are consistent and complementary, so that it is plausible to improve the ST resolution from the spot-level to cell-level by integrating the image information.

As a proof of concept, we compared the histological features and spatial transcriptomic features in the following samples, ductal carcinoma in situ (DCIS; 10x Genomics Visium data), human heart failure tissue with myocardial infarction (sample name: GT_IZ_P9_rep2 from paper[1]; 10x Genomics Visium data), mouse olfactory bulb (MOB; 10x Genomics Visium data), and the hippocampus region in mouse brain cerebellum (MERFISH data). The detailed sample information can be found in the Data Availability section in the main text. We extracted the histological features from H&E staining image patches surrounding the identified nuclei and subsequently applied principal component analysis (PCA) to capture essential variations within the histological features. Specifically, for the DCIS tissue, human heart failure tissue and mouse olfactory bulb tissue, the following nine image features were extracted: mean and standard deviation of the pixel colors intensities (red, green, blue, and gray), as well as the entropy of the image patches. For the mouse brain cerebellum tissue, the following six histological features were used: cell volume, mean and standard deviation of the gray color, logarithm of mean and standard variation of the gray intensity, and the entropy of the image patches. For obtaining the spatial transcriptomic features, we applied PCA to the expression levels of highly variable genes.

First, we show that at individual feature level, the histological and transcriptomic features capture cell organizations in a tissue. On the one hand, the first two principal components (PCs) of the histological features exhibit distinct spatial patterns (Supplementary Figure 29a). Specifically, in the tumor region of the DCIS tissue, high PC1 values marks cells near the boundary of the tumor regions and high PC2 values generally marks the interior of tumor regions (Supplementary Figure 29b). On the other hand, gene expression levels carry essential information about the cell type and cell-cell interaction in the tissue. For example, expression levels of *CD4* and *TDP52* can reflect the immune activity and the invasion of carcinoma in the tissue (Supplementary Figure 29c). The spatial expression patterns of *CD4* and *TDP52* in general agree with the patterns captured by PC1 and PC2 of histological features, respectively.

Next, we move on to cluster the cells/spots based on all the histological features or all the transcriptomic features. We clustered the cells using the PCA embeddings from the histological features with the Leiden algorithm[2]. The major cell clusters based on the histological features categorizes cells in regions such as tumor interior, boundary, and necrotic regions (Supplementary Figure 29d). Similarly, we also applied PCA analysis to the spatial transcriptomics data, and projection to the top 10 principal components were included for Leiden clustering. The spot clusters capture more patterns due to the high dimensionality in ST yet at the spot resolution. Across all the four samples, generally the dissection of tissue regions (layers) by major cell clusters based on merely the histological features agrees with the pattern based on merely the transcriptomic features (Supplementary Figures 29d-e).

References

1. Kuppe, C. et al. Spatial multi-omic map of human myocardial infarction. *Nature* **608**, 766-777 (2022).
2. Traag, V.A., Waltman, L. & van Eck, N.J. From Louvain to Leiden: guaranteeing well-connected communities. *Sci Rep* **9**, 5233 (2019).
