# Supplementary material for: Thor: a platform for cell-level investigation of spatial transcriptomics and histology
Source: Nat Commun. 2025 Aug 5;16:7178. doi: 10.1038/s41467-025-62593-1 (PMC12325965; doi:10.1038/s41467-025-62593-1)
Supplement: Supplementary file 5 — Description of additional supplementary files [file 41467_2025_62593_MOESM5_ESM.pdf]

DESCRIPTION OF ADDITIONAL SUPPLEMENTARY FILES

File: SupplementaryData1.xlsx
Supplementary Data 1
Caption: Gene signatures for cell types in mouse olfactory bulb (MOB) data.

File: SupplementaryData2.xlsx
Supplementary Data 2
Caption: Differentially expressed genes between cells in the fibrotic and non-fibrotic regions.

File: SupplementaryData3.xlsx
Supplementary Data 3
Caption: Gene signatures for cell types in ductal carcinoma in situ (DCIS) data.
